# National Survey of United Kingdom Paediatric Allergy Services

**DOI:** 10.1111/cea.14198

**Published:** 2022-08-03

**Authors:** Rosy Wells, Cathy McKay, Nick Makwana, Deepan Vyas, Sophie Vaughan, Anne Christopher, Lucy Thomas, Misbah Primett, Lavanya Diwakar, Shamir Alvis, Michael R. Perkin

**Affiliations:** ^1^ St. George's, University Hospitals NHS Foundation Trust London UK; ^2^ St. George's, University of London, Population Health Research Institute London UK; ^3^ Sandwell and West Birmingham Hospitals NHS Trust Birmingham UK; ^4^ West Hertfordshire Hospitals NHS Trust Watford UK; ^5^ Royal Stoke University Hospital Stoke‐on‐Trent UK; ^6^ Barts Health NHS Trust London UK

**Keywords:** clinical immunology, drug allergy, food allergy, immunotherapy, pediatrics, tolerance induction

## Abstract

**Background:**

Comprehensive national assessments of paediatric allergy services are rarely undertaken, and have never been undertaken in the United Kingdom. A 2006 survey estimated national capacity at 30,000 adult or paediatric new allergy appointments per year and identified 58 hospital clinics offering a paediatric allergy service.

**Objective:**

The UK Paediatric Allergy Services Survey was the first comprehensive assessment of UK paediatric allergy service provision.

**Methods:**

All 450 UK hospitals responded to a survey. Paediatric allergy services are provided in 154 lead hospitals with 75 further linked hospitals. All 154 lead paediatric allergy services completed a detailed questionnaire between February 2019 and May 2020.

**Results:**

The 154 paediatric allergy services self‐define as secondary (126/154, 82%) or tertiary (28/154, 18%) level services. The annual capacity is 85,600 new and 111,400 follow‐up appointments. Fifty‐eight percent (85/146) of services offer ≤10 new appointments per week (no data provided from 8 services—2 no response, 6 unknown) and 50% (70/139) of the services undertaking challenges undertake ≤2 food or drug challenges per week (no data from 3 challenge services). Intramuscular adrenaline is rarely used during challenges—median annual frequency 0 in secondary services and 2 in tertiary services. Allergen‐specific immunotherapy is offered in 39% (60/154) of services, with 71% (41/58) of these centres treating ≤10 patients per annum (no data from 2 immunotherapy services). The 12 largest services see 31% of all new paediatric allergy appointments, undertake 51% of new immunotherapy patient provision and 33% of food or drug challenges. Seventy percent (97/126) of secondary and all tertiary services are part of a regional paediatric allergy network. Only nine services offer immunotherapy for any food (3 for peanut), 10 drug desensitization and 18 insect venom immunotherapy.

**Conclusions:**

There has been a fourfold increase in paediatric allergy clinics and an approximately sevenfold increase in new patient appointment numbers in the United Kingdom over the past 15 years. Most services are small, with significant regional variation in availability of specific services such as allergen immunotherapy. Our findings emphasize the need for national standards, local networks and simulation training to ensure consistent and safe service provision.

Key Messages

- Paediatric allergy services increased fourfold between 2006 and 2020 in the United Kingdom.
- Most paediatric allergy services are relatively small, suggesting a need for national standards and networking.
- There is significant regional variation in provision of some paediatric allergy services such as allergen immunotherapy.

## 1 INTRODUCTION

The atopic conditions asthma, eczema, allergic rhinitis, anaphylaxis, conjunctivitis, food allergy and urticaria/angioedema collectively affect over one in three children in the United Kingdom and are estimated to cost the NHS over £1 billion per annum.^1^ The United Kingdom has one of the highest rates of allergic disease and whilst the prevalence of hay fever and eczema has plateaued or decreased,^2^ in contrast admissions for anaphylaxis, food allergy, urticaria and angioedema have increased significantly.^2^, ^3^ Commencing in 2003 with the seminal report by the Royal College of Physicians “Allergy: the Unmet Need”, a series of subsequent reports have emerged highlighting concerns about the paucity of paediatric allergy services (reviewed in detail in the Supplementary Appendix). The British Society of Allergy and Clinical Immunology (BSACI) undertook a survey of allergy services for a House of Commons report in 2006.^4^ This was based on data from the BSACI website clinic finding service, but this is neither complete nor up to date.^5^ We have therefore undertaken the first comprehensive survey of every UK hospital to establish which are providing a paediatric allergy service and what that service consists of. The intention of the survey is to also act as a repository so that health professionals and patients and their families can identify the location of services appropriate to their needs. Furthermore, by identifying areas where inconsistencies exist, the information may be used to help drive publication of national standards for paediatric allergy services, similar to those in adult allergy services.^6^ The results will also allow individual services to benchmark themselves against other paediatric allergy services and may help inform decisions regarding the structure and development of services and networks.

## 2 METHODS

**FIGURE 1:**
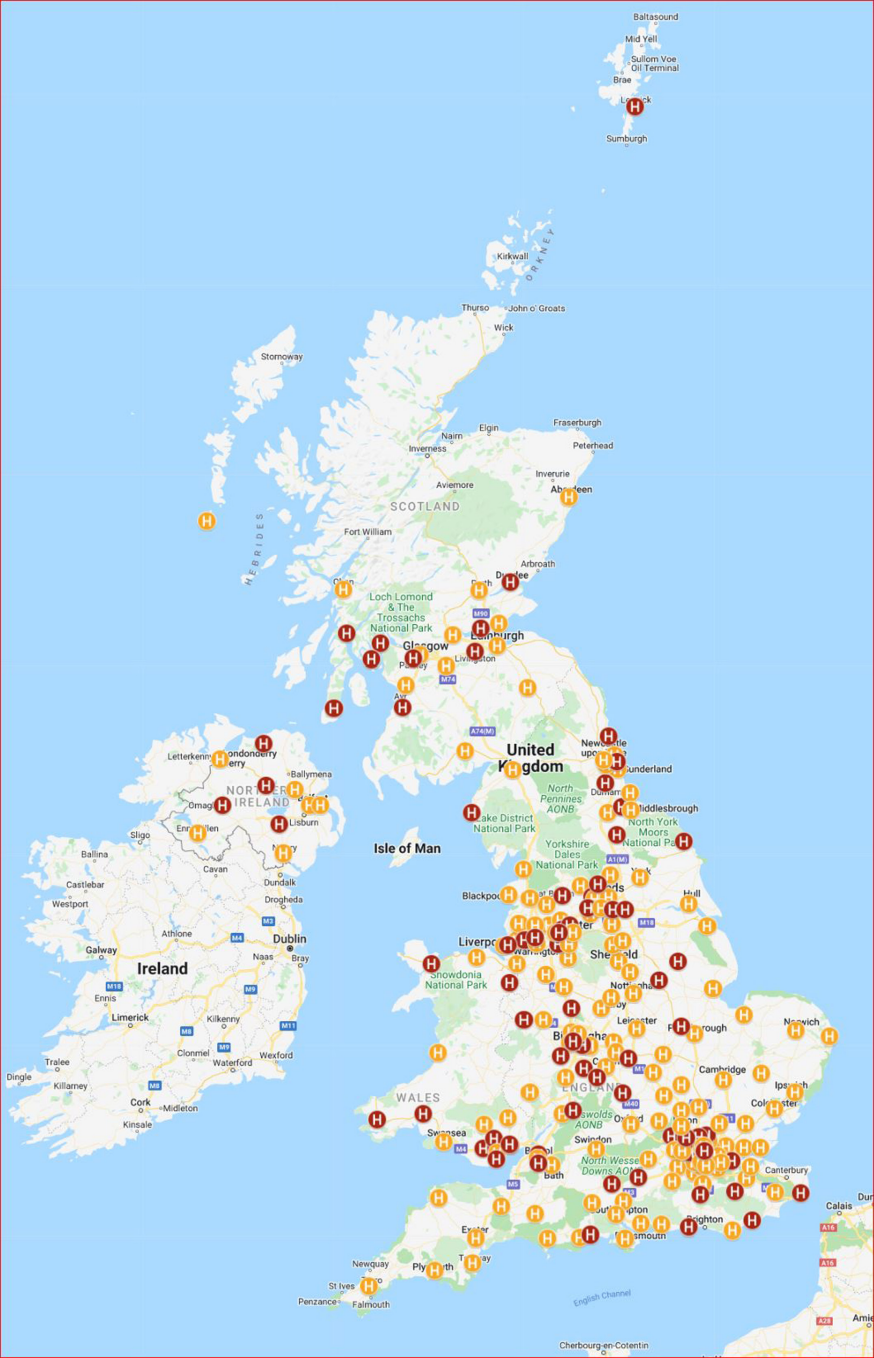
UK Paediatric Allergy Services ‐ Configuration map. The UK Paediatric Allergy Services ‐ Configuration Map webpage includes a Map Legend within which ten different “layers” can be selected: Paediatric allergy services (denoting the Lead and Link paediatric allergy services in the UK); Allergy Network membership (specific network); Nurses undertaking Allergy Consultations (i.e. instead of a doctor); Dieticians undertaking Allergy Consultations (i.e. instead of a doctor); Dietician Support; Joint Clinic ‐ Gastroenterology; Joint Clinic ‐ Dermatology; Joint Clinic ‐ Respiratory; Adolescent Clinic; Transition Clinic. In this Figure, the layer Paediatric Allergy Services has been selected and Lead services are indicated in orange and Linked services in red. https://doi.org/10.24376/rd.sgul.20292489

The survey was completed in two stages. In the first stage, the contact details of all 450 hospitals in the United Kingdom were found from online searches. Each hospital was contacted to establish whether the hospital provides a paediatric allergy service. If a paediatric allergy service is provided, the respondent was asked for the contact details of the person best placed to answer more detailed questions about the service. The 450 hospitals are part of 173 trusts. All 450 hospitals responded and 229 reported providing a paediatric allergy service. Some trusts provide paediatric allergy services in more than one hospital. The lead hospital was identified, resulting in 154 hospitals where the paediatric allergy service is principally provided (lead service) and 75 other hospitals where the service provided is linked to the provision in a lead hospital service (linked service) (Figure 1). A detailed questionnaire was then sent to the lead hospitals (questionnaire available in the Supplementary Material). Details of how the questionnaire was constructed and the rationale behind what questions to include are discussed in the Supplementary Appendix.

## 3 RESULTS

All 154 lead services seeing paediatric allergy patients nationally completed the survey between February 2019 and May 2020. The two last respondents completed their surveys after the start of the Covid‐19 pandemic and were asked to record the configuration of their service prior to any impact of the pandemic. Respondents were asked if they provide a secondary or tertiary allergy service or both. The terms were not further defined and interpretation was entirely at the discretion of the respondent. Responses were as follows: 126 (82%) secondary only and 3 (2%) tertiary only and 25 (16%) secondary and tertiary level paediatric allergy care. For subsequent analyses, tertiary only and secondary and tertiary respondents were combined. Seventy‐one (46%) were providing paediatric allergy services in one or more other hospitals.

### 3.1 Map of UK paediatric allergy service provision

Based on the questionnaire response data we have produced three interactive online maps showing the configuration, investigations and treatments undertaken by the 154 hospitals seeing paediatric allergy patients. The maps, intended for use by healthcare practitioners and the public, are available at: https://doi.org/10.24376/rd.sgul.20292489.

### 3.2 Structure and staffing configuration (Table 1)

**TABLE 1:** Location, structure and staffing

	Secondary services (*N* = 126)	Tertiary services (*N* = 28)	All services
	% (*n*/*N*)	% (*n*/*N*)	% (*n*/*N*)
Staffing and configuration			
Medical staff seeing paediatric allergy patients			
General paediatrician	31 (39/126)	36 (10/28)	32 (49/154)
General paediatrician with subspecialty interest (≥50%)	25 (31/126)	46 (13/28)	29 (44/154)
General paediatrician with subspecialty interest (<50%)	71 (89/126)	57 (16/28)	68 (105/154)
Subspecialist paediatrician	7 (9/126)	54 (15/28)	16 (24/154)
Adult immunologist	2 (3/126)	11 (3/28)	4 (6/154)
Associate specialist	19 (24/126)	14 (4/28)	18 (28/154)
Subspecialty interest of General Paediatricians with a special interest			
Allergy	91 (97/107)	100 (24/24)	92 (121/131)
Respiratory	62 (66/107)	38 (9/24)	57 (75/131)
Dermatology	14 (15/107)	21 (5/24)	15 (20/131)
Gastroenterology	19 (20/107)	21 (5/24)	19 (25/131)
Immunology	7 (7/107)	17 (4/24)	8 (11/131)
Other^c^	13 (14/107)	13 (3/24)	13 (17/131)
Subspecialty interest of Paediatric Subspecialists			
Allergy	67 (6/9)	93 (14/15)	83 (20/24)
Respiratory	44 (4/9)	33 (5/15)	38 (9/24)
Dermatology	11 (1/9)	27 (4/15)	21 (5/24)
Gastroenterology	22 (2/9)	20 (3/15)	21 (5/24)
Immunology	11 (1/9)	40 (6/9)	29 (7/24)
Other^d^	11 (1/9)	27 (4/15)	21 (5/24)
Formal training of consultants contributing to paediatric allergy service			
Postgraduate certificate in allergy	24 (30/126)	21 (6/28)	23 (36/154)
MSc in allergy	21 (27/126)	46 (13/28)	26 (40/154)
MD/PhD in allergy	4 (5/126)	32 (9/28)	9 (14/154)
SPIN training in allergy	9 (11/126)	21 (6/28)	11 (17/154)
GRID training in allergy	2 (2/126)	43 (12/28)	9 (14/154)
EAACI accredited paediatric allergist	2 (2/126)	29 (8/28)	6 (10/154)
Other allergy training experience^e^	61 (77/126)	39 (11/28)	57 (88/154)
None	11 (14/126)	4 (1/28)	10 (15/154)
Service configuration			
Designated lead for the service^a^	78 (98/125)	96 (27/28)	82 (125/153)
All consultants contributing to paediatric allergy service have ≥2 Pas in job plan^a^	71 (89/126)	46 (13/28)	66 (102/154)
At least one designated paediatric allergy nurse^a^	89 (104/116)	96 (27/28)	91 (131/144)
Nurses have formal paediatric allergy training^b^	35 (36/104)	37 (10/27)	35 (46/131)
Paediatric dietitian available to support patients^a^	92 (115/125)	100 (28/28)	93 (143/153)
Runs at least one paediatric allergy clinic per week^a^	83 (104/126)	93 (26/28)	89 (137/154)
≥30 minutes for new patient appointment^a^	84 (102/122)	81 (22/27)	83 (124/149)
Clinics coded as 255 or 420^a^	72 (46/64)	80 (16/20)	74 (62/84)

^a^ Secondary Care BSACI Standard.

^b^ Specialist Paediatric Allergy Service Standards.

^c^ Other: Neonatology (*n* = 4), community paediatrics (*n* = 2), emergency medicine, infectious diseases, rheumatology, nephrology, ENT.

^d^ Other: ENT, infectious diseases.

^e^ Other: Diploma in allergy, allergy training days (regional or national), clinical/training experience, attendance at conferences, working towards formal qualifications, and other formal qualifications (FRCPath Immunology, Denver National Jewish Training, MSc/PG Cert in related subspecialties).

Most paediatric allergy services are small. The median number of consultant whole time equivalents (WTE) providing services specifically to paediatric allergy services is 0.5 (range 0–14) in secondary level services and 1.5 (range 0.2–6.1) in tertiary services. Whilst dietician support is present in most paediatric allergy services (92% secondary and all tertiary), WTE dietician provision is very limited (0.1 WTE in secondary and 0.5 WTE in tertiary). Total annual service capacity amounts to 85,600 new paediatric allergy appointments (51,000 in secondary level services and 34,600 in tertiary services) and 111,400 follow‐up paediatric allergy appointments in the United Kingdom. Further details of paediatric allergy service structure and configuration are presented in the Supplementary Appendix and Supplementary Figures S1–S3.

### 3.3 Allergy investigations (Table 2)

**TABLE 2:** Investigations and diagnostics

	Secondary Services (*N* = 126)	Tertiary Services (*N* = 28)	All services
	% (*n*/*N*)	% (*n*/*N*)	% (*n*/*N*)
Investigations			
Specific IgE	100 (126/126)	100 (28/28)	100 (154/154)
Skin prick testing	95 (120/126)	100 (28/28)	96 (148/154)
Component resolved diagnostic testing^b^	71 (89/126)	89 (25/28)	74 (114/154)
ISAC macro‐array testing	21 (27/126)	46 (13/28)	26 (40/154)
Intradermal testing	5 (6/126)	68 (19/28)	16 (25/154)
Patch testing	2 (3/126)	7 (2/28)	3 (5/154)
Spirometry^a^	59 (74/126)	79 (22/28)	62 (96/154)
Exhaled nitric oxide^b^	25 (32/126)	54 (15/28)	31 (47/154)
Other^c^	1 (1/126)	11 (3/28)	3 (4/154)
Skin prick testing			
Skin prick testing available on same day^a^	93 (111/120)	96 (27/28)	93 (138/148)
Skin testing for drugs, food, venom and latex^b^	9 (11/120)	46 (13/28)	16 (24/148)
Staff undertaking skin prick testing			
Consultant	31 (37/120)	14 (4/28)	28 (41/148)
Associate specialist	3 (3/120)	4 (1/28)	3 (4/148)
Nurse	91 (109/120)	100 (28/28)	93 (137/148)
Specialist registrar	2 (2/120)	4 (1/28)	2 (3/148)
Laboratory technician	1 (1/120)	0 (0/28)	1 (1/148)
Dietician	4 (5/120)	14 (4/28)	6 (9/148)
Other^d^	7 (8/120)	0 (0/28)	5 (8/148)
Skin prick testing undertaken			
Foods—commercial SPT solutions	99 (119/120)	100 (28/28)	99 (147/148)
Foods—fresh whole foods	93 (111/120)	100 (28/28)	94 (139/148)
Aeroallergens	93 (112/120)	96 (247/28)	94 (139/148)
Latex	71 (85/120)	86 (24/28)	74 (109/148)
Bee/wasp venom	26 (31/120)	54 (15/28)	31 (46/148)
Drugs	18 (22/120)	68 (19/28)	28 (41/148)
Other^e^	0 (0/120)	4 (1/28)	1 (1/148)
Skin prick testing to whole foods			
Tahini (sesame)	54 (59/110)	64 (18/28)	56 (77/138)
Fresh cow's milk	62 (68/110)	61 (17/28)	62 (85/138)
Raw egg white	30 (33/110)	32 (9/28)	30 (42/138)
Family brought food	93 (102/110)	96 (27/28)	93 (129/138)
Nuts	38 (42/110)	50 (14/28)	41 (56/138)
Component testing			
Peanut	98 (86/88)	96 (23/24)	97 (109/112)
Hazelnut	74 (65/88)	83 (20/24)	76 (85/112)
Venom	17 (15/88)	75 (18/24)	29 (33/112)
Birch	42 (37/88)	88 (21/24)	52 (58/112)
Other	33 (29/88)	63 (15/24)	39 (44/112)
Peanut specific components (if peanut component testing undertaken)			
ara h1	56 (48/85)	48 (11/23)	55 (59/108)
ara h2	98 (83/85)	100 (23/23)	98 (106/108)
ara h3	40 (34/85)	39 (9/23)	40 (43/108)
ara h8	87 (74/85)	78 (18/23)	85 (92/108)
ara h9	51 (43/85)	48 (11/23)	50 (54/108)
Hazelnut specific components (if hazelnut component testing undertaken)			
cor a1	73 (45/62)	90 (18/20)	77 (63/82)
cor a8	77 (48/62)	80 (16/20)	78 (64/82)
cor a9	84 (52/62)	75 (15/20)	82 (67/82)
cor a14	84 (52/62)	80 (16/20)	83 (68/82)
Food & Drug challenges			
Provides food challenges^b^	90 (114/126)	100 (28/28)	92 (142/154)
Provides drug challenges^b^	44 (56/126)	96 (27/28)	54 (83/154)
Staff undertaking drug challenges			
Consultant	45 (25/56)	59 (16/27)	49 (41/83)
Associate specialist	7 (4/56)	4 (1/27)	6 (5/83)
Specialist registrar	7 (4/56)	26 (7/27)	13 (11/83)
Allergy nurse specialist	50 (28/56)	78 (21/27)	59 (49/83)
Paediatric nurse	43 (24/56)	33 (9/27)	40 (33/83)
Dietician	0 (0/56)	0 (0/27)	0 (0/83)
Other^f^	9 (5/56)	0 (0/27)	6 (5/83)
Drug challenges undertaken			
Analgesics—paracetamol	55 (31/56)	85 (23/27)	65 (54/83)
Analgesics—NSAIDs	55 (31/56)	81 (22/27)	64 (53/83)
Antibiotics—IV	5 (3/56)	56 (15/27)	22 (18/83)
Antibiotics—oral	98 (55/56)	100 (27/27)	99 (82/83)
Local anaesthetics	11 (6/56)	70 (19/27)	30 (25/83)
General anaesthetics^b^	2 (1/56)	44 (12/27)	16 (13/83)
Other^g^	4 (2/56)	19 (5/27)	8 (7/83)
Open food challenge location			
Paediatric day ward	80 (90/113)	71 (20/28)	78 (110/141)
Dedicated challenge unit	1 (1/113)	21 (6/28)	5 (7/141)
Outpatient department	8 (9/113)	11 (3/28)	9 (12/141)
Inpatient ward	20 (23/113)	4 (1/28)	17 (24/141)
Supervised feeds location			
Paediatric day ward	73 (30/41)	53 (9/17)	67 (39/58)
Dedicated challenge unit	0 (0/41)	29 (5/17)	9 (5/58)
Outpatient department	20 (8/41)	41 (7/17)	26 (15/58)
Inpatient ward	17 (7/41)	12 (2/17)	16 (9/58)
Staff undertaking food challenges			
Consultant	25 (28/114)	39 (11/28)	27 (39/142)
Associate specialist	5 (6/114)	7 (2/28)	6 (8/142)
Specialist registrar	4 (5/114)	18 (5/28)	7 (10/142)
Allergy nurse specialist	39 (45/114)	64 (18/28)	44 (63/142)
Paediatric nurse	69 (79/114)	57 (16/28)	67 (95/142)
Dietician	3 (3/114)	4 (1/28)	3 (4/142)
Other^h^	7 (8/114)	4 (1/28)	6 (9/142)
Challenge service configuration			
Resuscitation facilities available for challenge^a^	98 (112/114)	100 (28/28)	99 (140/142)
≥2 challenges performed per month^a^	99 (110/111)	100 (28/28)	99 (138/139)
Supervision of ≤2 challenges per nurse^a^	89 (93/105)	82 (23/28)	87 (116/133)
Written information provided for challenges^a^	86 (98/114)	93 (26/28)	87 (124/142)
Parental consent obtained for challenges^a^	69 (78/113)	82 (23/28)	72 (101/141)
Challenge database kept^a^	64 (73/114)	86 (24/28)	68 (97/142)

^a^ Secondary Care BSACI Standard.

^b^ Specialist Paediatric Allergy Service Standards.

^c^ Other: Tertiary—basophil activation tests (BAT tests) (*n* = 1), ocular testing including visual acuity, intraocular pressure, staining and anterior rhinoscopy (*n* = 1), exercise challenge test (*n* = 2). Secondary—patch testing of standard dermatology panel (*n* = 1).

^d^ Other: includes healthcare assistants and ENT staff.

^e^ Other: includes major and minor determinants.

^f^ Other: includes junior doctor (with support) and clinical support worker.

^g^ Other: steroids, immunosuppressants, biologicals, codeine and “all” drugs.

^h^ Other: includes nurse practitioner, nurse clinician, associate practitioner, healthcare assistant and Senior House Officer.

All services offer specific IgE testing and 95% (147/154) undertake skin prick testing. Component resolved diagnostics (CRD) is offered by 71% (89/126) of secondary services and 89% (25/28) of tertiary services. Intradermal testing, exhaled nitric oxide, spirometry and ISAC panels are more frequently offered at tertiary centres. Patch testing for foods is offered in five allergy services, two tertiary and three secondary.

Logistics of skin prick testing including staff undertaking it, allergens tested, products used and technical aspects of skin prick test interpretation are reviewed in detail in the Supplementary Appendix.

#### 3.3.1 Intradermal testing

Of the 25 services offering intradermal testing to paediatric allergy patients, six are secondary and 19 are tertiary services. Intradermal testing is most commonly offered to local anaesthetic agents (5 secondary, 19 tertiary), general anaesthetics (2 secondary, 14 tertiary) and antibiotics (3 secondary, 14 tertiary). Wasp and bee venom intradermal testing are offered by 13 services (all tertiary). Intradermal testing to other drugs was listed by three tertiary services and to chlorhexidine by one tertiary service.

#### 3.3.2 Component resolved diagnostics (CRD) testing

Three‐quarters of respondents use CRD (114/154). Of the 112 respondents who reported the individual components used, 97% (109) test peanut components, 76% (85) use hazelnut components, 29% (33) venom components (Wasp Ves v5, Bee Api m1), 52% (58) birch (Bet v1 and homologues) and 39% (44) use other components. Of individuals testing for peanut components, 10% (11/109) test components in all suspected peanut‐allergic children. For individuals testing selected patients, the most common reasons given to use component testing for peanut are as follows: to differentiate patients with pollen food syndrome/oral allergy syndrome (22), to assess severity of allergy (4), where there is diagnostic uncertainty (18) and prior to oral food challenges (9). One individual reported that the decision to perform component testing is made in the laboratory. Ara h2 is the most routinely tested component (98%, 106/108), followed by ara h8 (85%, 92/108), ara h1 (55%, 59/108), ara h9 (50%, 54/108) and ara h3 (40%, 43/108). Hazelnut components are tested routinely on all children with suspected hazelnut allergy by 9% (8/85) of respondents. Reasons for testing were similar to peanut components. Hazelnut components (cor a1, cor a8, cor a9 and cor a14) are routinely tested with similar frequency.

#### 3.3.3 Drug challenges

Drug challenges are much more common in tertiary services (96% vs. 44%). Allergy nurse specialists (59%, 49/83), consultants (49%, 41/83) and paediatric nurses (40%, 33/83) most commonly perform drug challenges. Oral antibiotic challenges are offered by 99% (82/83) of centres offering drug allergy challenges. Paracetamol or NSAID challenges are offered in over half of secondary services where drug challenges are undertaken and the majority of tertiary centres. In contrast, challenges to IV antibiotics and local and general anaesthetics take place almost exclusively in tertiary centres.

#### 3.3.4 Food challenges

Ninety percent(114) of secondary and all (28) tertiary services perform food challenges, almost all as open challenges (secondary 97%, 111/114 and tertiary 96%, 27/28). Supervised feeds are more commonly offered in tertiary services (61%, 17/28) as opposed to secondary (38%, 43/114). Double‐blind challenges are rarely offered in secondary services (8%, 9/114) compared with tertiary (50%, 14/28). Just over half of services undertake some form of risk stratification for their challenges (61% of secondary, 69/114 and 57% of tertiary, 16/28).

The median number of challenges undertaken per week is as follows: secondary 2 (111), interquartile range 1–4, range 0.2–17 and tertiary 8 (28), IQR 4–18, range 1–55. Hence many centres are performing low numbers of challenges: 50% (70/139) of centres perform ≤2 challenges per week. An estimated 677 challenges are therefore being performed per week across the 139 centres reporting their average number of challenges. Assuming challenges take place 45 weeks per year this suggests that 30,474 challenges are performed per year in food‐allergic children in the United Kingdom. Details of how challenges are configured and their staffing are presented in the Supplementary Appendix.

#### 3.3.5 Food challenge practice

Written information about the challenge process for parents and children is provided by most services: secondary 86% (98/114) and tertiary 93% (26/28). Written consent prior to the challenge is not universal, but more common in tertiary services (82%, 23/28) than secondary (69%, 78/113). In contrast, a starting lip dose was much more likely to be used in secondary care (68%, 78/114) than tertiary (39%, 11/28).

Nearly all services offer baked egg challenges (98%, 137/140) or baked milk challenges (94%, 131/140). Raw egg challenges are less frequently offered and more commonly in tertiary services (36%, 10/28) than secondary (25%, 28/112).

#### 3.3.6 Food challenge outcomes

A database of challenges undertaken is more commonly maintained in tertiary services (86%, 24/28) than secondary (64%, 73/114). The use of a standardized protocol for recording symptoms and signs during a challenge (e.g. PRACTALL) is similar between levels of service (secondary 60%, 68/114 and tertiary 61%, 17/28). Intramuscular adrenaline use during challenges in the previous year is remarkably infrequent. The median annual frequency of administration is as follows: secondary 0, IQR 0–1, range 0–10 and tertiary 2, IQR 1–8.5, range 0–42. Hence 61% of secondary services (67/110) and 21% of tertiary services (6/28) have not administered a single dose of intramuscular adrenaline during a challenge in the preceding year.

Respondents were asked to estimate what proportion of food challenges during the preceding year were positive. For standard risk food challenges the median percentage estimated to be positive was as follows: secondary (101) 20%, IQR 10%–26%, range 0%–90% and tertiary 20% (27), IQR 16%–30%, range 10%–50%. For high risk food challenges the median percentage positive was as follows: secondary 20% (47), IQR 5%–45%, range 0%–100%, and tertiary 28% (12), IQR 17.5%–45%, range 0%–50%. For supervised feeds the corresponding positive figures were as follows: secondary 5% (31), IQR 0%–20%, range 0%–50%, and tertiary 9% (15), IQR 1%–15%, range 0%–50%. Food challenges were most likely to be coded as a day case admission for a procedure (secondary 73%, 82/112, tertiary 89%, 25/28). Twelve percent (13/112) of secondary services and 7% (2/28) of tertiary services did not know how challenges were coded.

### 3.4 Treatments, management and clinical governance

#### 3.4.1 Allergen Immunotherapy

Thirty‐nine percent (60/154) of services offer allergen‐specific immunotherapy for allergic rhinitis, much more frequently in tertiary services (86%, 24/28) than secondary (29%, 36/124). Eight hundred and ninety‐two new patients are offered immunotherapy per year by the 58/60 centres reporting numbers. A few tertiary centres offer the majority of immunotherapy: 71% (41) of centres offer immunotherapy to ≤10 new patients per year and only 14% (9) centres offer immunotherapy to 20 or more patients per year. Immunotherapy numbers are capped in one‐third (19/60) of services.

Allergens to which paediatric allergy services offer immunotherapy are as follows: grass (60), tree (28), house dust mite (22) and pets (7). Among services offering immunotherapy, sublingual immunotherapy (SLIT) is available in all of the 36 secondary services, but only 83% (20/24) of the tertiary services. Subcutaneous immunotherapy (SCIT) is offered in 4 secondary and 19 tertiary services. No services offer epicutaneous immunotherapy or intralymphatic immunotherapy. Commercial products used for SLIT are shown in Table 3. SLIT and SCIT appointments are most commonly coded as day cases (55%, 29/53 and 73%, 16/22 respectively). Eighty‐three percent (50/60) of services maintain a database of children undergoing immunotherapy and 77% (46/60) obtain written consent for patients undergoing immunotherapy treatment. For services using Grazax, over half (25/48) of services report that the GP takes over the funding for this. In services that reported the time funding was taken over, this was most commonly after 1 month (47%, 9/19).

**TABLE 3:** Treatments, management and governance

	Secondary Services (*N* = 126)	Tertiary Services (*N* = 28)	All services
	% (*n*/*N*)	% (*n*/*N*)	% (*n*/*N*)
Treatments			
Immunotherapy offered			
Grass	29 (36/126)	86 (24/28)	39 (60/154)
Tree	7 (9/126)	68 (19/28)	18 (28/154)
House dust mite	6 (7/126)	54 (15/28)	14 (22/154)
Pets	1 (1/126)	21 (6/28)	5 (7/154)
Other^c^	0 (0/126)	7 (2/28)	1 (2/154)
Form of immunotherapy offered			
Sublingual immunotherapy^b^	29 (36/126)	71 (20/28)	36 (56/154)
Subcutaneous immunotherapy^b^	3 (4/126)	68 (19/28)	15 (23/154)
Epicutaneous immunotherapy	0 (0/126)	0 (0/28)	0 (0/154)
Intralymphatic immunotherapy	0 (0/126)	0 (0/28)	0 (0/154)
Sublingual immunotherapy product offered			
Lofarma Allergoid sublingual immunotherapy (LAIS)	6 (2/35)	15 (3/20)	9 (5/55)
Oralvac compact	20 (7/35)	70 (14/20)	38 (21/55)
Oraltek	0 (0/35)	5 (1/20)	2 (1/55)
Grazax	91 (32/35)	90 (18/20)	91 (50/55)
Acarizax	9 (3/35)	25 (5/20)	15 (8/55)
Subcutaneous immunotherapy product offered			
Pollinex	100 (4/4)	47 (9/19)	57 (13/23)
Pollinex Quattro	50 (2/4)	63 (12/19)	61 (14/23)
Alutard SQ	0 (0/4)	5 (1/19)	4 (1/23)
Allergovit	25 (1/4)	16 (3/19)	17 (4/23)
Acaroid	25 (1/4)	11 (2/19)	13 (3/23)
Novo‐Helisen Depot	0 (0/4)	5 (1/19)	4 (1/23
Other^d^	0 (0/4)	5 (1/19)	4 (1/23
Immunotherapy service configuration			
Immunotherapy database maintained^a^	75 (27/36)	96 (23/24)	83 (50/60)
Management			
Reintroduction ladder usage			
Home introduction of well cooked (baked) egg			
IgE type allergy (no asthma or anaphylaxis)	79 (83/105)	67 (16/24)	77 (99/129)
IgE type allergy (asthma but no anaphylaxis)	33 (35/105)	38 (9/24)	34 (44/129)
IgE type allergy (anaphylaxis)	1 (1/105)	4 (1/24)	2 (2/129)
Non‐IgE type allergy	81 (85/105)	92 (22/24)	83 (107/129)
Reintroduction ladder not used for this	2 (2/105)	4 (1/24)	2 (3/129)
Home introduction of lightly cooked egg (if tolerating well cooked egg)			
IgE type allergy (no asthma or anaphylaxis)	68 (71/104)	58 (14/24)	66 (85/128)
IgE type allergy (asthma but no anaphylaxis)	30 (31/104)	42 (10/24)	32 (41/128)
IgE type allergy (anaphylaxis)	3 (3/104)	8 (2/24)	4 (5/128)
Non‐IgE type allergy	73 (76/104)	88 (21/24)	76 (97/128)
Reintroduction ladder not used for this	8 (8/104)	4 (1/24)	7 (9/128)
Home introduction of raw egg			
IgE type allergy (no asthma or anaphylaxis)	28 (28/100)	32 (7/22)	29 (35/122)
IgE type allergy (asthma but no anaphylaxis)	13 (13/100)	18 (4/22)	14 (17/122)
IgE type allergy (anaphylaxis)	2 (2/100)	0 (0/22)	2 (2/122)
Non‐IgE type allergy	40 (40/100)	55 (12/22)	43 (52/122)
Reintroduction ladder not used for this	54 (54/100)	41 (9/22)	52 (63/122)
Home introduction of dairy using iMAP ladder			
IgE type allergy (no asthma or anaphylaxis)	53 (63/120)	54 (15/28)	53 (78/148)
IgE type allergy (asthma but no anaphylaxis)	26 (31/120)	32 (9/28)	27 (40/148)
IgE type allergy (anaphylaxis)	1 (1/120)	0 (0/28)	1 (1/148)
Non‐IgE type allergy	92 (110/120)	100 (28/28)	93 (138/148)
Reintroduction ladder not used for this			
Desensitization offered			
Venom^b^	4 (5/121)	46 (13/28)	12 (18/149)
Food	2 (3/121)	21 (6/28)	6 (9/149)
Drug^b^	2 (2/121)	29 (8/28)	7 (10/149)
Not provided	93 (113/121)	43 (12/28)	84 (125/149)
Food desensitization offered			
Peanut	0 (0/3)	50 (3/6)	33 (3/9)
Milk	100 (3/3)	67 (4/6)	78 (7/9)
Egg	33 (1/3)	50 (3/6)	44 (4/9)
Patient support and training			
Patient information leaflets offered			
Locally designed	48 (61/126)	79 (22/28)	54 (83/154)
Allergy UK	79 (99/126)	71 (20/28)	77 (119/154)
Anaphylaxis Campaign	50 (63/126)	54 (15/28)	51 (78/154)
From drug companies	20 (25/126)	29 (8/28)	21 (33/154)
From other paediatric allergy centres	21 (27/126)	18 (5/28)	21 (32/154)
Other^e^	25 (32/126)	36 (10/28)	27 (42/154)
Not provided	2 (2/126)	0 (0/28)	1 (2/154)
Patient, parent and carer training offered			
AAI training^a^	96 (121/126)	100 (28/28)	97 (149/154)
Inhaler use^a^	82 (103/126)	86 (24/28)	82 (127/154)
Eczema management^a^	60 (76/126)	86 (24/28)	65 (100/154)
Nasal spray/drop use^a^	69 (87/126)	96 (27/28)	74 (114/154)
Other^f^			
No training offered			
Management plans available for all patients^a^	95 (120/126)	100 (28/28)	96 (148/154)
Governance			
≥Once monthly MDTs^a^	38 (47/125)	75 (21/28)	44 (68/153)
Part of a regional network^a^	77 (97/126)	100 (28/28)	81 (125/154)
Linked with tertiary centre^a^	42 (52/125)	n/a	n/a
≥1 educational event for GPs per year^a^	69 (87/126)	86 (24/28)	72 (111/154)

^a^ Secondary Care BSACI Standard.

^b^ Specialist Paediatric Allergy Service Standards.

^c^ Other: includes horse and peanut.

^d^ Other: Pharmalgen (Bee/Wasp) and Tyrosin TU.

^e^ Other: includes British Association of Dermatologists (BAD), BSACI, British Dietetic Association (BDA), Children and Young's People Allergy Network Scotland (CYANS), Regional Allergy Network information leaflets, Itchy, Sneezy, Wheezy Project.

^f^ Other: Lifestyle choices/advice, School training, Asthma management (more broadly).

#### 3.4.2 Omalizumab

Omalizumab is offered to treat severe urticaria in 25 centres: 7% (9/125) of secondary and 57% (16/28) of tertiary services.

#### 3.4.3 Reintroduction Ladders

Ninety‐eight percent (149/152) of services use reintroduction ladders; all (100%, 149/149) use the milk ladder and 87% (129/149) use the egg ladder. An egg reintroduction ladder is most commonly used for home introduction of well‐cooked egg for children with non‐IgE mediated allergy (83%, 107/129) and IgE mediated allergy to egg with no asthma or anaphylaxis (77%, 99/129). Milk reintroduction ladder (iMAP) is most commonly used for milk introduction in non‐IgE mediated allergy (93%, 138/148) and IgE mediated milk allergy with no asthma or previous anaphylaxis (53%, 78/148).

#### 3.4.4 Desensitization Programmes

Desensitization is offered by 16% (24/149) of services. Eighteen services offer desensitization to insect venom (5 secondary, 13 tertiary), 9 to food (3 secondary, 6 tertiary) and 10 to drugs (2 secondary, 8 tertiary). For services offering food desensitization, peanut is offered by 3, milk by 7 and egg by 4 centres.

#### 3.4.5 Allergy reaction management

Twenty‐two percent (34/152) of services prescribe only one brand of adrenaline autoinjector (AAI). Epipen is most commonly prescribed (86%, 131/152), followed by Emerade (73%, 111/152) and Jext (73%, 111/152). Most services (79%,120/154) issue cetirizine, 16% (24/154) chlorphenamine and 6% (9/152) issue another antihistamine, most commonly loratadine/desloratadine. Respondents completing the survey on a paper copy usually ticked more than one option (this was not possible on the online survey), suggesting that many more respondents would have reported prescribing more than one antihistamine had the option been available. Eighty‐three percent (128/154) of centres issue BSACI management plans for allergic reactions. Twenty percent (31/154) provide locally designed management plans and 5% (8/154) use management plans from other centres. Three percent (5) of centres do not issue management plans.

#### 3.4.6 Patient support and training

Patient information sheets are sourced primarily from Allergy UK (77%, 119/154), locally designed information (54%, 83/154) and the Anaphylaxis Campaign (51%, 78/154). AAI training is the most common form of training offered to patients, parents or carers (97%, 149/154).

#### 3.4.7 Clinical governance

**FIGURE 2:**
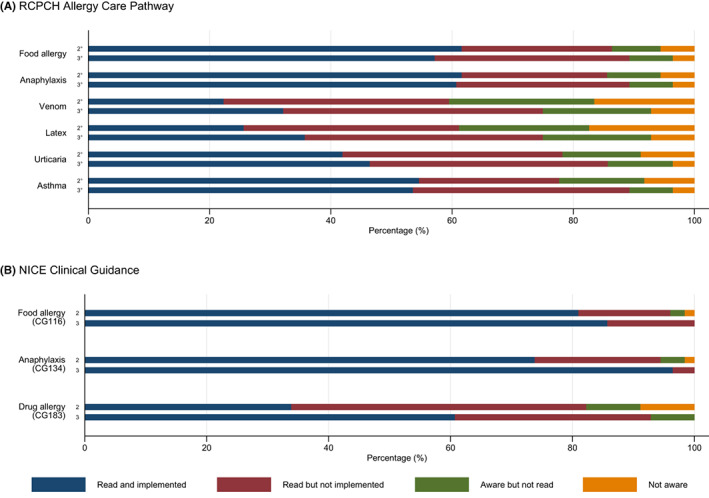
Royal College of Paediatrics and Child Health Care Pathways (Panel A) and National Institute for Clinical Excellence Guidance (Panel B). Data shown are whether for the respective RCPCH Care Pathway or NICE Clinical Guidance the respondent has: Read and implemented the document (Navy); Read but implemented the document (Red); Aware but not read the document (Green); or Not aware of the document (Orange).

NICE guidelines have been read and implemented by a larger percentage of services than RCPCH Allergy Care Pathways (Figure 2). Fifty‐six percent (70/125) of secondary services and 89% (25/28) of tertiary services hold MDTs. This is most commonly on a monthly basis (39%, 27/69) in secondary services and weekly (52%, 13/25) in tertiary services.

Seventy‐seven percent (97/126) of secondary and 100% (28/28) of tertiary services are part of a regional paediatric allergy network. Nearly half (42%, 52/125) of secondary services are linked to a tertiary centre.

Eighty‐five percent (131/154) of services offer paediatric allergy educational events, most commonly for colleagues (90%, 118/131) and GPs (87%, 114/131). Teaching for colleagues is given most commonly every 6 months (45%, 52/115) and annually or less for GPs (64%, 72/112).

#### 3.4.8 Follow‐up and transition arrangements

Fifty‐five percent (84/154) of services have a routine frequency of follow‐up for paediatric allergy patients, with patterns being broadly similar between secondary and tertiary services (Supplementary Figure S4). Seven percent (11/154) of services run an adolescent clinic and 8% (13/154) have a transition clinic. An adult allergy service is offered by 29% (44/153) of hospitals. When patients exceed the age threshold for paediatric allergy services, 35% (53/153) of respondents discharge all patients back to GP, 7% (11/153) refer all patients to adult services and 58% (89/153) refer some on to adult services. Patients referred on are referred to: adult allergy (91%, 90/99), dermatology (25%, 25/99), respiratory (38%, 38/99) and other services (9%, 9/99) including immunology (4%, 4/99), ENT (3%, 3/99), gastroenterology (3%, 3/99) and ophthalmology (3%, 3/99).

#### 3.4.9 BSACI secondary care and specialist paediatric allergy service standards

Few secondary care services fully adhere to all the secondary care standards set out by BSACI^7^ and similarly few tertiary services to all the service specification standards for paediatric allergy specialist centres published by NHS England^8^ (Tables 1, 2, 3).

#### 3.4.10 Regional variation in paediatric allergy service provision

**FIGURE 3:**
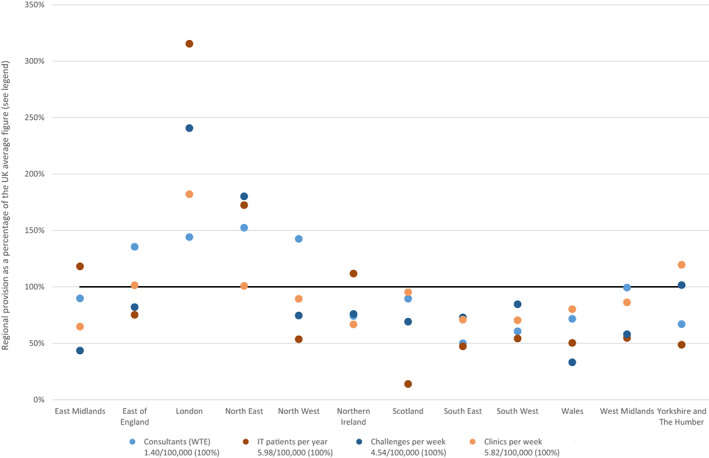
Regional differences in paediatric allergy services. Data shown are the regional differences in four metrics: (1) The average number of consultants (WTE) providing paediatric allergy services (Light blue), (2) New immunotherapy patients per year (Red), (3) Challenges undertaken per week (Navy) and (4) Paediatric allergy clinics undertaken per week (Yellow) The provision of each metric per 100,000 children (0–17 years) in the United Kingdom is shown in the legends. For each region the provision per 100,000 children in that region was calculated and this is shown as a percentage of the UK figure. For example, there are 208 WTE consultants proving paediatric allergy services in the United Kingdom to 14,908,442 children. This equates to 1.40 WTE consultants per 100,000 children (the UK average). East Midlands has 13.3 WTE consultants for 1,060,868 children, that is, 1.25 per 1000,000 children. This is 90% of the UK average figure and hence is plotted at 90%.

Each region of the United Kingdom has one or more self‐designated tertiary centres with the exception of Northern Ireland. London predominates with seven tertiary centres. However, marked differences remain in core features of paediatric allergy service provision per 100,000 children in the different regions (Figure 3). London is above the UK average for all the metrics whilst the South East and Wales are significantly below. Scotland fares particularly poorly on provision of immunotherapy to children with the lowest availability per 100,000 children in the United Kingdom.

## 4 DISCUSSION

### 4.1 Service capacity

There has clearly been a proliferation in the number of hospitals across the United Kingdom offering a paediatric allergy service. The 154 lead services and 75 link services surveyed together contribute to a very significant expansion in paediatric allergy appointment capacity which now stands at 85,600 new and 111,400 follow‐up appointments per year. This is a marked increase on the BSACI 2006 figure of 30,000 new allergy appointments for adults and children per year, 6750 specifically in paediatric allergy clinics and the rest in adult clinics, some of which also saw children.^4^ The data, therefore, suggest an approximate‐ sevenfold increase in paediatric new allergy appointment capacity since the 2006 survey (assuming 20% of the patients seen in adult clinics in 2006 were children). Similarly, in 2006 BSACI recorded 58 paediatric allergy clinics and we identified 229 in 2019/2020, which represents a fourfold increase in numbers. An analysis of how current paediatric allergy consultant provision compares with that reported in the Allergy: the Unmet Need report is included in the Supplementary Appendix.

### 4.2 Small services predominate

Clinic capacity is heavily skewed towards smaller services with one in four secondary services and one in five tertiary services undertaking only one clinic per week seeing exclusively paediatric allergy patients. There are no requirements within either the BSACI standards for secondary care or the NHS contract for specialist paediatric allergy services with regards to the number of patients that must be seen within the service, beyond stating for the former that the service should be undertaking at least one allergy clinic per week. The consequence of many services being small is that they undertake very low numbers of challenges and, in general, there was very little reported use of intramuscular adrenaline. This raises a concern about confidence in managing anaphylaxis. Further investigation as to whether this reflects under usage of intramuscular adrenaline, or selection of very low‐risk patients is warranted.

Three‐quarters of all secondary services and all tertiary services are part of a local network. This has the potential benefit of allowing sharing of guidelines, protocols, teaching, education and support. However, it needs to be determined whether being part of a network results in improvements in paediatric allergy care.

### 4.3 Provision of multidisciplinary paediatric allergy services

With regards to specific staffing of paediatric allergy services, dietetic input is essential for the management of paediatric allergy patients^9^ and BSACI secondary standards state that a paediatric dietician should be available and competent to support patients with food allergy.^7^ Eight percent of services do not have access to dietetic support. Nearly half of services reported that children are referred to a dietician from allergy clinic, meaning a separate visit/appointment for a dietetic appointment, as well as a delay in support and advice for food‐allergic children and their families. A “one‐stop shop” model provides a cost‐effective and more convenient service for children and their families.^10^

### 4.4 Diagnostics capacity

Thirty thousand four hundred and sixty‐five challenges are performed per year in food‐allergic children in the United Kingdom. This number has to meet the needs of those children from the outpatient consultations who require a challenge and as well as children already known to the paediatric allergy service requiring challenges. We did not specifically ask about waiting list for challenges, but from our own experience, and discussion with peers, low capacity and long waiting lists for challenges is commonplace across allergy services. Significantly, pressure could potentially ensue from the multiple‐day visits required for Palforzia, the newly licensed therapy for peanut immunotherapy, and from the increasing provision needed for selective nut introduction and early introduction challenges.

### 4.5 Therapeutics capacity

Allergen immunotherapy for allergic rhinitis is indicated for children with moderate‐to‐severe AR that is otherwise uncontrolled despite pharmacotherapy.^11^ Of the 8,402,154 children 5–16 years (ONS 2016) in the United Kingdom, approximately 34,000 would be eligible for SIT and current provision is 892 new patients per year. This is a very significant increase on the 323 children treated over 10 years (32 per year) in Vance's study. Eight hundred and ninety‐two new patients per year over 10 years represents 26% of the 34,000 eligible children and contrasts with Vance's estimate of 1%. Furthermore, there has been a clear improvement in the number of services providing immunotherapy with 60 declared, albeit that most of these are doing a very small number of cases. This concurs with the BRIT report which stated that “Most immunotherapy is limited to specialist allergy centres in the United Kingdom,”^12^ and indeed, from our survey only nine centres offer immunotherapy to 20 or more patients per year and availability of immunotherapy for food, drug or insect stings was limited.

### 4.6 Significant variation in practice

Variation was observed in all aspects of paediatric allergy service provision including skin prick testing methodology (compared with the BSACI^13^ and WAO^14^ guidelines), the use of whole foods for SPT (discussed further in the Supplementary Material), challenge practice with respect to risk stratification, staff ratios, emergency support, and data collection and use of reintroduction ladders out with the use for which the ladders were designed. Five allergy centres do not offer training with AAIs and further clarity about whether this is provided in the community or via the pharmacy is required. Paediatric allergy services see patients with asthma, eczema and allergic rhinitis as well as food allergies and, as per BSACI secondary standards, all services should be expected to offer training in use of inhalers, eczema management and nasal spray technique.^7^ Many services are not providing patients and carers with this support.

### 4.7 Regional provision for less common allergy‐related activity

The aspiration for there to be at least one regional centre providing the full spectrum of specialized paediatric allergy services remains appropriate but is not being fulfilled. The number of services offering highly specialized diagnostic and treatment facilities remains low, for example diagnostic testing for and investigation of drug allergy, particularly to intravenous antibiotics and local and general anaesthetics.

Few services offer adolescent and/or transition clinics. EAACI recently published guidelines for the management of adolescents and young adults with allergy and asthma that will help ensure ensure that services are providing for this group of children, either within existing clinics or in separate clinics.^15^

### 4.8 Strengths and limitations of study

The strength of this survey is the 100% response rate from all services seeing paediatric allergy patients across the United Kingdom, providing the first comprehensive assessment paediatric allergy service provision. The principal limitation is that the responses to the survey are unverified and hence our interpretations of the data are dependent on the accuracy of the responses. Furthermore, for some questions, the response given in the survey reflects an individual clinician's personal practice and may not reflect the practice of other colleagues within a service. We also did not include private practitioners offering paediatric allergy services. In some parts of the country, such provision is significant. However, the paediatric allergy training of individuals providing private paediatric allergy services is often minimal to non‐existent and the quality of care provided is extremely variable. The data from this survey are contributing to an ongoing international comparison of paediatric allergy services.

## 5 CONCLUSION

In summary, there has been a welcome increase in paediatric allergy service provision, but much of this is provided in small services. Increased clinical case exposure brings with it increased clinical experience. The need for quality standards for paediatric allergy services, similar to those produced recently for adult allergy services, endorsed by both the Royal College of Paediatrics and Child Health (RCPCH) and BSACI, is recognized. Quality standards already exist for other paediatric subspecialties, such as gastroenterology, and the production of similar standards and accreditation for paediatric allergy will ensure that children with allergic diseases receive high‐quality and consistent care, regardless of their geographical location.

## AUTHOR CONTRIBUTIONS

Michael R Perkin & Rosy Wells substantially contributed to design, concept, acquisition of data, analysis and interpretation of data, drafting the article, final approval of the version to be published and agreed to be accountable for all aspects of the work. Cathy McKay and Shamir Alvis substantially contributed to acquisition of data and final approval of the version to be published. NM and DV substantially contributed to design, interpretation of data and final approval of the version to be published. Sophie Vaughan, Anne Christopher, Lucy Thomas, Misbah Primett and Lavanya Diwakar substantially contributed to design and final approval of the version to be published.

## Funding information

RW received funding from Wellcome Trust ISSF Academic Collaboration Fund.

## CONFLICT OF INTEREST

The authors have no conflicts of interest to declare.

## ETHICAL STATEMENT

Ethical approval was not required for this service evaluation.

## Supporting information


Appendix S1

Appendix S2

## Data Availability

The data that support the findings of this study are openly available at: https://doi.org/10.24376/rd.sgul.20292489.
