# Supplementary material for: National Survey of United Kingdom Paediatric Allergy Services
Source: Clin Exp Allergy. 2022 Aug 3;52(11):1276–90. doi: 10.1111/cea.14198 (PMC9804618; doi:10.1111/cea.14198)
Supplement: Supplementary file 1 — Appendix S1 [file CEA-52-1276-s002.pdf]

bsaci
improving allergy care
through education, training and research

UK Paediatric Allergy Services Survey

In our previous Freedom of Information request, your trust was declared to be providing paediatric allergy services.

As a follow up Freedom of Information request, we are very grateful for you providing further information about these services.

We very much hope the results of this survey will help to contribute to improving the provision of paediatric allergy services in the UK.

The survey takes 30-45 mintues to complete. Please note the instructions to omit sections that don't apply to your paediatric allergy service. The expectation is that you will be able to answer the questions without having to seek additional information about your service. In places, we ask you to prove an estimate for your response - absolute precision is not necessary.

Paediatric allergy services

Q1 What level of paediatric allergy service are you providing?

- ◯ Secondary only
- ◯ Tertiary only
- ◯ Both Secondary and Tertiary

Q2 What is the name of **the trust** you are responding on behalf of?

[ ]

Q3 Does your trust, provide paediatric allergy services in more than one hospital?

- ◯ Yes
- ◯ No

Q4 Please write the name of **each hospital** where paediatric allergy services are provided by your trust:

[ ]

Paediatric allergy services in primary care

Q5 Have you established paediatric allergy services in primary care?

- ☐ No special link established
- ☐ Paediatric allergy staff from the trust go into primary care to offer a paediatric allergy clinic/service
- ☐ GPs have been trained to provide a paediatric allergy service in primary care
- ☐ Other provision of allergy services in primary care

If other provision of allergy services in primary care, please describe [ ]

The following series of questions ask about the staffing configuration of the paediatric allergy services that are provided. The first questions are about senior medical staffing, followed by junior medical staffing, nursing staffing and then dietican support.
Please answer this series of questions assuming you are fully staffed, i.e. consider a vacant post as being filled

Medical Staffing

Q6 Which medical staff undertake clinics in your trust where paediatric allergy patients are seen? (please choose all that apply)

- ☐ Consultant General Paediatricians
- ☐ Consultant General Paediatricians with a subspeciality interest (≥50% of time with paediatric allergy patients)
- ☐ Consultant General Paediatricians with a subspeciality interest (<50% of time with paediatric allergy patients)
- ☐ Consultant Subspecialist Paediatricians
- ☐ Adult Immunologists
- ☐ Associate Specialists

Q7 *If your paediatric allergy service includes Consultant General Paediatricians with a subspeciality interest:* What are the subspeciality interests of the **Consultant General Paediatricians with a subspeciality interest** involved in seeing paediatric allergy patients (please choose all that apply)

- ☐ Allergy Interest
- ☐ Respiratory Interest
- ☐ Dermatology Interest
- ☐ Gastroenterology Interest
- ☐ Immunology Interest
- ☐ Other Interest (please specify below)

Other Consultant General Paediatrician interest (please specify) ______

Q8 *If your paediatric allergy service includes Consultant Subspecialist Paediatricians:*What are the subspeciality interests of the **Consultant Paediatric Subspecialists** involved in seeing paediatric allergy patients (please choose all that apply)

- ☐ Allergy
- ☐ Respiratory
- ☐ Dermatology
- ☐ Gastroenterology
- ☐ Immunology
- ☐ Other subspeciality (please specify below)

Other Consultant Paediatric Subspeciality (please specify) ______

Q9 What formal allergy training have consultants contributing to the service had (please choose all that apply)?

- ☐ None of the consultant staff have had formal allergy training
- ☐ Postgraduate Certificate in Allergy
- ☐ MSc in Allergy
- ☐ MD/PhD in Allergy Research
- ☐ SPIN training in Allergy
- ☐ GRID training in Allergy
- ☐ EAACI accredited Pediatric Allergist (Diploma)
- ☐ Other allergy training/experience

If other allergy training and/or experience, please describe ______

Q10 Do you have a designated lead for your Paediatric Allergy Service?

- ◯ Yes
- ◯ No

Q11 How many consultants contribute to seeing paediatric allergy patients?
NB this refers to the medical staffing you have listed previously i.e. consultant paediatricians with or without a subspeciality interest, paediatric subspecialists, immunologists and associate specialists. It is not referring to the extended medical team such as dermatologists, ENT consultants etc, or to paediatric subspecialists not listed in Q8, e.g. neonatologists, all of whom see patients with paediatric allergy problems.

Consultants (number of individuals) [ ]

Total (for all consultants) number of Whole Time Equivalents (WTE) of time providing services to paediatric allergy patients [ ]

Q12 Do all consultant staff working in your paediatric allergy service have a minimum of 2 PA's in their job plan designated for paediatric allergy?

- ◯ Yes
- ◯ No

Q13 Are the staff involved in seeing paediatric allergy patients members of the BSACI?
Ideally answer as e.g. 5 out of 7 are members

[ ]

Junior Medical Staffing

Q14 Do specialist registrars and/or clinical research fellows regularly attend the clinics where paediatric allergy patients are seen?

- ◯ Yes - they see patients independently
- ◯ Yes - sit in on clinic but do not see patients independently
- ◯ No

Nursing Staffing

*If your paediatric allergy service has no nursing support please skip this section and go to the next section headed "**Dietician support**" (Q22)*

Q15 How many nurses contribute to looking after paediatric allergy patients?

Nurses (number of nurses) [ ]

Total (for all nurses) number of Whole Time Equivalents (WTE) of time providing services to paediatric allergy patients [ ]

Q16 What band are the nurse/s who contribute to seeing paediatric patients?

Number who are Band 5 [ ]

Number who are Band 6 [ ]

Number who are Band 7 [ ]

Number who are Band 8 or more [ ]

Q18 *If you have any Band 8 nursing staff:* What role do your Band 8 nursing staff hold? (please choose all that apply)

- ☐ Nurse Consultant
- ☐ Advanced Nurse Practitioner
- ☐ Clinical Nurse Specialist
- ☐ Other

If other, please describe: [ ]

Q19 Allergy training of the nurse/s in the paediatric allergy service

- ◯ All nurse/s have had formal training in allergy
- ◯ Some nurse/s have had formal training in allergy
- ◯ No nurses have had formal training in allergy

Q20 Do nursing staff undertake independent nurse led clinics seeing paediatric allergy patients?

- ◯ Yes
- ◯ No

Q21 *If nursing staff are undertaking independent nurse led clinics:* In independent nurse led clinics, what kinds of paediatric allergy patients are seen?

- ☐ New paediatric allergy referrals (i.e. never been seen by a medical doctor within the allergy service)
- ☐ Follow up paediatric allergy appointments
- ☐ Paediatric allergy patients referred to the nurse led clinic for allergy testing or training
- ☐ Immunotherapy patient clinics (independently nurse led)

Dietician support

If your paediatric allergy service has no dietican support please skip this section and go to the next section headed "**Paediatric Allergy Clinics**" (Q25)

Q22 How much dietician support does your paediatric allergy service have?

- ◯ No dietician support
- ◯ Dietician present at all paediatric allergy clinics
- ◯ Dietician present at some paediatric allergy clinics
- ◯ Dietician not present but sees paediatric allergy patients by referral from the paediatric allergy service

Q23 How much dietician time do you think your paediatric allergy service has specifically for paediatric allergy patients? (in Whole Time Equivalents)
For example, if you undertake one morning allergy clinic per week and a dietician is always present then this would be 0.1 WTE dietician time

[ ]

Q24 Do dieticians undertake independent dietician led clinics seeing new paediatric allergy referrals?
i.e. referrals for a paediatric allergy consultation NOT specifically a paediatric dietician consultation

- ◯ Yes
- ◯ No

Paediatric Allergy Clinics

Q25 In what kind of clinic are paediatric allergy patients seen?

- ◯ In general paediatric clinics amongst other paediatric patients
- ◯ In clinics with exclusively paediatric allergy patients
- ◯ Both of the above

Q26 Approximately **how many clinics** does your paediatric allergy service undertake **per week**?

General clinics where paediatric allergy patients are seen amongst other paediatric patients? [ ]

Paediatric allergy clinics with exclusively paediatric allergy patients [ ]

Q27 Approximately what proportion (%) of the **general paediatric clinic** consultations, on average, would you say relate to paediatric allergy problems? (please give a percentage as an estimate)

[ ]

Q28 Approximately how many **paediatric allergy patients** does your service undertake outpatient consultations for per week?

New Outpatient appointments each week [ ]

Follow Up appointments each week [ ]

Q29 What is your **best estimate** of the waiting time for paediatric allergy patients new and follow up appointments? *Please **answer in months**, if no waiting list please enter 0*

New Outpatient appointment waiting time (months) [ ]

Follow Up appointment waiting time (months) [ ]

Joint Clinics

Q30 Does your paediatric allergy service undertake any joint clinics i.e. paediatric allergy with another speciality?

- ○ Yes
- ○ No

Q31 *If your paediatric allergy service offers joint clinics:* What joint clinics does your paediatric allergy service offer?

- ☐ Joint clinic with paediatric gastroenterology
- ☐ Joint clinic with paediatric respiratory
- ☐ Joint clinic with paediatric dermatology
- ☐ Joint clinic with other specialist

Joint clinic with other specialist, please specify who [ ]

Paediatric Allergy Clinic Configuration

Q32 When do the clinics occur when paediatric allergy patients are seen?

- ☐ Morning
- ☐ Afternoon
- ☐ Evening

Q33 *For the relevant clinics:* How long are the outpatient clinics in which paediatric allergy patients are seen?

Morning clinic (answer in hours please) [ ]

Afternoon clinic (answer in hours please) [ ]

Evening clinic (answer in hours please) [ ]

Paediatric Allergy Consultation Duration

For the next two questions, please provide answers for the staffing that apply to your paediatric allergy service. Please leave ones that do not apply empty.

Q34 How long is the *average* appointment time for a **NEW** paediatric allergy consultation? (please answer in minutes) *If you don't know the answer please enter a zero*

New patient appointment duration with the **Consultant** [ ]

New patient appointment duration with the **Associate Specialist** [ ]

New patient appointment duration with the **Allergy Nurse Specialist** [ ]

Average new patient appointment duration with the **Specialist Registrar** [ ]

Average new patient appointment duration with the **Dietician** [ ]

Q35 How long is the *average* appointment time for a **FOLLOW UP** paediatric allergy consultation? (please answer in minutes)
If you don't know the answer please enter a zero

Follow up appointment duration with the **Consultant** [ ]

Follow up appointment duration with the **Associate Specialist** [ ]

Follow up appointment duration with the **Allergy Nurse Specialist** [ ]

Follow up appointment duration with the **Specialist Registrar** [ ]

Follow up appointment duration with the **Dietician** [ ]

Q36 *If you undertake general paediatric clinics where paediatric allergy patients are seen:* Please describe how your service templates **general clinics** where paediatric allergy patients are seen amongst other paediatric patient?

Morning clinic: Number of **new** patients [ ]

Morning clinic: Number of **follow up** patients [ ]

Afternoon clinic: Number of **new** patients [ ]

Afternoon clinic: Number of **follow up** patients [ ]

Q37 *If you undertake specialist paediatric allergy clinics:* Please describe how your service templates clinics **seeing exclusively paediatric allergy patients**?

Morning clinic: Number of **new** patients [ ]

Morning clinic: Number of **follow up** patients [ ]

Afternoon clinic: Number of **new** patients [ ]

Afternoon clinic: Number of **follow up** patients [ ]

Q38 How is a paediatric allergy outpatient appointment in your paediatric allergy service coded?

- [ ] As 255 Paediatric Clinical Immunology and Allergy
- [ ] As 317 Allergy
- [ ] As 420 Paediatrics
- [ ] Don't know
- [ ] Other

If Other, please specify [ ]

Q39 Do you know, even if only approximately, what your trust is reimbursed per patient for the paediatric allergy services it provides?

- ( ) Yes
- ( ) No

Q40 If known: How much does your trust get reimbursed for the following paediatric allergy services?

New patient consultation (£) [ ]

Follow up consultation (£) [ ]

Day case attendance (£) [ ]

Allergy Investigations

Q41 What diagnostic tests are offered in your paediatric allergy service? (please choose all that apply)

- [ ] Blood tests (specific IgE/RAST tests)
- [ ] Skin Prick tests
- [ ] Component resolved diagnostic tests
- [ ] ISAC ImmunoCAP test
- [ ] Intradermal tests
- [ ] Patch testing for foods
- [ ] Spirometry
- [ ] Exhaled nitric oxide measurement
- [ ] Other

If Other, please specify ______

Skin Prick Testing

If skin prick testing is not undertaken in your paediatric allergy service please skip this section and go to the next section headed **"Intradermal Tests**" (Q54)

Q42 Are skin prick tests usually undertaken on the same day or in a separate clinic on a different day?

- ○ Same day
- ○ Different day

Q43 Who regularly performs skin tests at your clinic (please choose all that apply)

- [ ] Consultant
- [ ] Associate specialist
- [ ] Nurse
- [ ] Specialist registrar
- [ ] Lab technician
- [ ] Dietician
- [ ] Other

If Other, please specify ______

Q44 Do you have resuscitation facilities immediately available for skin tests?

- ○ Yes
- ○ No

Q45 What do you undertake <u>skin prick testing</u> to?

- [ ] Foods - commercial skin prick test solutions
- [ ] Foods - using fresh whole foods
- [ ] Aeroallergens
- [ ] Latex
- [ ] Bee/wasp venom
- [ ] Drugs
- [ ] Other

If Other skin prick testing undertaken, please specify ______

Q46 Which commercial skin prick solutions do you use? *(More than one may be ticked)*

- ☐ Soluprick (ALK)
- ☐ Allergy Therapeutics
- ☐ Immunotek
- ☐ Diagenics (Allergopharma)
- ☐ Other

If Other SPT solution used, please state ______

Q47 If you use whole foods for testing: Which whole foods to you use for skin prick testing?

- ☐ Tahini (sesame)
- ☐ Fresh cow's milk
- ☐ Raw egg white
- ☐ Other food brought in by the patient
- ☐ Nuts

If Nuts, please state which ______

Q48 If you are using whole foods for testing: For the foods that you are using for skin prick testing, do you use them alone (instead of the commercial solution), or as well as the commercial solution

	Whole food only	Whole food and commercial solution at same time
Sesame	○	○
Fresh cow's milk	○	○
Raw egg white	○	○
Nuts if entered in Q47	○	○

Q49 How does your service measure skin prick test wheal size?

- ○ Largest diameter of the wheal
- ○ Mean of the of largest diameter of the wheal and its perpendicular diameter

Q50 Do you deduct the size of the negative control from the allergen responses, if the negative control response is positive?

- ○ Yes
- ○ No

Q51 What do you consider a positive skin prick test response to an allergen?

- ○ ≥ 3 mm diameter
- ○ Any positive response

Q52 Do you include the diameter of psuedopods when measuring the largest diameter?

- ○ Yes
- ○ No

Q53 Do you have a threshold that the histamine positive control has to exceed to consider it valid and hence the tests to be interpretable?

- ○ Yes
- ○ No

If yes, what size does the histamine response have to be greater or equal to in millimetres? ☐

Intradermal tests

Q54 If you undertake intradermal testing: What do you offer intradermal testing to?

- ☐ Antibiotics
- ☐ Local anaesthetics
- ☐ General anaesthetics
- ☐ Bee/wasp venom
- ☐ Other

If Other intradermal testing offered, please state to what? [ ]

Component testing

If component testing is not undertaken please skip this section and go to the next section headed "**Challenge Tests**" (Q60)

Q55 What do you undertake component testing to?

- ☐ Peanut components
- ☐ Hazelnut components
- ☐ Venom components (Wasp Ves v5, Bee Api m1)
- ☐ Birch components (Bet v1 and homologues)
- ☐ Other components

Q56 If you undertake peanut component testing: Who do you measure peanut components on?

- ○ All suspected peanut allergic children
- ○ Specific children

Please describe what criteria determine who you chose to measure peanut components on [ ]

Q57 If you undertake peanut component testing: Which peanut components do you **routinely** measure, if you chose to measure them?

- ☐ ara h1
- ☐ ara h2
- ☐ ara h3
- ☐ ara h8
- ☐ ara h9

Q58 If you undertake hazelnut component testing: Who do you measure hazelnut components on?

- ○ All suspected hazelnut allergic children
- ○ Specific children

Please describe what criteria determine who you chose to measure hazelnut components on [ ]

Q59 If you undertake hazelnut component testing: Which hazelnut components do you **routinely** measure, if you chose to measure them?

- ☐ cor a1
- ☐ cor a8
- ☐ cor a9
- ☐ cor a14

Challenge tests

Q60 Are food and/or drug challenges offered at your service?

◯ Food challenges only	Please go to section headed "**Food challenges**" (Q63)
◯ Food and drug challenges	Please go to next question (Q61)
◯ No challenges undertaken	Please go to section headed **"Immunotherapy"** (Q84)

Drug challenges

Q61 Who performs drug challenge tests at your service (please choose all that apply)

- ☐ Consultant
- ☐ Associate specialist
- ☐ Specialist registrar
- ☐ Allergy Nurse specialist
- ☐ Paediatric Nurse
- ☐ Dietician
- ☐ Other

If Other, please specify ______

Q62 What classes of drugs do you offer challenges to ?

- ☐ Analgesics - paracetamol
- ☐ Analgesics - NSAIDs
- ☐ Antibiotics - IV
- ☐ Antibiotics - oral
- ☐ Local anaesthetic agents
- ☐ General anaesthetic agents
- ☐ Other

If Other, please specify ______

Food challenges

Q63 What type of food challenges do you undertake? (please choose all that apply)

- ☐ Open food challenges
- ☐ Supervised feeds
- ☐ Blinded food challenges

Q64 Do you stratify your open food challenges into high risk and standard risk challenges?

- ◯ Yes
- ◯ No

Q65 Where do you undertake your challenges? (please choose all that apply)

	Paediatric day ward	Dedicated challenge unit	Outpatients	Inpatient ward
Open food challenges	☐	☐	☐	☐
Supervised feeds	☐	☐	☐	☐

Q66 Do you have resuscitation facilities immediately available for challenges?

- ◯ Yes
- ◯ No

Challenge service configuration

Q67 On average, how many challenges do you undertake per week in your service?

[ ]

Q68 How many challenges are undertaken in one session?
If the challenges come in at staggered times, please give the total for the session, e.g. a hospital may do 8 challenges in one session, 4 at 9am and 4 at 11am, the answer here would be 8.

[ ]

Q69 How do you configure the challenges that are done in one session?
Please describe how this number of challenges is configured: all arrive at same time? Or staggered - please describe as per the example above.

[ ]

Q70 How many patients undergoing challenges are supervised concurrently by each individual nurse or doctor?

Open food challenges [ ]

Open food challenges - high risk [ ]

Supervised feeds [ ]

Q71 If some challenges are designated high risk: How does the challenge configuration differ for challenges designated high risk, compared to low/standard risk? Please explain

[ ]

Challenge test staffing

Q72 Who performs food challenge tests at your service (please choose all that apply)

- ☐ Consultant
- ☐ Associate specialist
- ☐ Specialist registrar
- ☐ Allergy Nurse specialist
- ☐ Paediatric Nurse
- ☐ Dietician
- ☐ Other

If Other, please specify [ ]

Q73 Does your service undertake nurse **led** challenges?

- ○ Yes
- ○ No

Q74 *If nursing staff are undertaking nurse **led** challenges:* Is any specific cover arranged for nurse led challenges?

- ○ Junior doctors provide cover for clerking, consent and treatment of reactions
- ○ Junior doctors present on unit but not directly involved with challenges
- ○ Doctor providing paediatric allergy services not present but scheduled to be available if needed
- ○ Junior doctor not present but specifically scheduled to be available if needed
- ○ General paediatric on call team specifically scheduled to be available if needed
- ○ No specific cover arranged - general paediatric on call team could be called if needed
- ○ Other arrangement

If Other arrangement, please specify [ ]

Challenge tests practice

Q75 Do you provide written information for parents and children about the challenge process?

- ○ Yes
- ○ No

Q76 Do you obtain written consent to undertake challenges?

- ○ Yes
- ○ No

Q77 Do you start your food challenges with a lip dose (rubbing a small amount on the inner part of the child's lips and then observing the child)?

- ○ Yes
- ○ No

Q78 Do you offer challenges to the following foods (please choose all that apply)?

- ☐ Baked egg
- ☐ Baked milk
- ☐ Raw egg white

Challenge tests outcomes

Q79 Do you maintain a database of children undergoing challenge procedures and their outcomes?

- ○ Yes
- ○ No

Q80 Do you record symptoms/signs during a challenge using a standardised protocol?

- ○ Yes - use a standardised protocol
- ○ No - have own symptom/sign recording protocol

If yes, whose protocol (e.g. a tertiary hospital's or a published protocol such as PRACTALL) [ ]

Q81 Approximately how many times has intramuscular adrenaline had to be administered to paediatric allergy patients in the last year?

[ ]

Q82 Over the course of a year, please estimate what percentage of your challenges are positive? (e.g. if 1 in 3, please write 33)

Standard risk challenges: % positive [ ]

High risk challenges: % positive [ ]

Supervised feeds: % positive [ ]

Q83 How is an appointment for a challenge test coded in your service?

- ○ As a day case admission for procedure
- ○ As an outpatient appointment
- ○ As an inpatient admission
- ○ Unknown
- ○ Other

If Other, please specify [ ]

Immunotherapy

Q84 Do you provide allergen specific immunotherapy for allergic rhinitis (pollen/HDM/pets)?

- ◯ Yes
- ◯ No

If immunotherapy is not provided, please go to the section headed "**Omalizumab (Xolair)**" (Q96)

Q85 Which allergens do you offer immunotherapy to? (please choose all that apply)

- ☐ Grass
- ☐ Tree
- ☐ House dust mite
- ☐ Pets
- ☐ Other

If Other, please specify

Q86 Is the number of people you provide immunotherapy to capped each year?

- ◯ Yes
- ◯ No

Q87 Approximately, how many new patients do you offer immunotherapy to each year?

Q88 What form of immunotherapy do you undertake? (please choose all that apply)

- ☐ Sub-lingual immunotherapy (SLIT)
- ☐ Sub-cutaneous immunotherapy (SCIT)
- ☐ Epicutaneous immunotherapy (EPIT)
- ☐ Intralymphatic immunotherapy

Q89 If you undertake SLIT: Which products do you use for your sublingual immunotherapy (SLIT)? (please choose all that apply)

- ☐ LAIS (Lofarma Allergoid Sublingual Immunotherapy) (Manufacturer Lofarma, Italy; UK distributor Captium Limited)
- ☐ Oralvac compact (Manufacturer Allergy Therapeutics, UK)
- ☐ Oraltek (Manufacturer Immunotek, Spain; UK distributor Bio-Diagnostics Ltd)
- ☐ Grazax (Manufacturer ALK)
- ☐ Acarizax (Manufacturer ALK)
- ☐ Other SLIT product

If Other SLIT product, please specify

Q90 If you undertake SCIT: Which products do you use for your subcutaneous immunotherapy (SCIT)? (please choose all that apply)

- ☐ Pollinex (Manufacturer Allergy Therapeutics, UK)
- ☐ Pollinex Quattro (Manufacturer Allergy Therapeutics, UK)
- ☐ Alutard SQ (Manufacturer ALK)
- ☐ Allergovit (Manufacturer Allergopharma, Diagenics)
- ☐ Acaroid (Manufacturer Allergopharma, Diagenics)
- ☐ Novo-Helisen Depot (Manufacturer Allergopharma, Diagenics)
- ☐ Other SCIT product

If Other SCIT product, please specify

Q91 How is an appointment for immunotherapy coded in your service?

	As day case	As outpatient appointment	As inpatient admission	Unknown	Other
Sub-lingual (SLIT)	◯	◯	◯	◯	◯
Subcutaneous (SCIT)	◯	◯	◯	◯	◯

Q92 If Other form of coding used for immunotherapy, please specify

Q93 Do you maintain a database of children undergoing immunotherapy?

- ◯ Yes
- ◯ No

Q94 Do you obtain written consent for patients undergoing immunotherapy?

- ◯ Yes
- ◯ No

Q95 If you use Grazax: Do you ask GPs to take over the funding of Grazax?

- ◯ Yes
- ◯ No

If yes, after how long? (answer in months please)

Omalizumab (Xolair)

Q96 Do you offer omalizumab (Xolair) therapy for severe urticaria in your clinic?

- ◯ Yes
- ◯ No

Reintroduction ladders

Q97 Do you use reintroduction ladders for the following foods?

- ☐ Egg (e.g. BSACI guidelines for the management of egg allergy - egg ladder)
- ☐ Milk (e.g. iMAP/MAP)
- ☐ Don't use

Q98 If you use reintroduction ladders:In what situation do you use the reintroduction ladder

	IgE type allergy (no asthma or anaphylaxis)	IgE type allergy (asthma but no anaphylaxis)	IgE type allergy (anaphylaxis)	Non-IgE type allergy	Don't use a reintroduction ladder for this
Home introduction of well cooked (e.g. baked) egg	☐	☐	☐	☐	☐
Home introduction of lightly cooked egg (if tolerating well cooked egg)	☐	☐	☐	☐	☐
Home introduction of raw egg	☐	☐	☐	☐	☐
Home introduction of dairy using iMAP/MAP ladder	☐	☐	☐	☐	☐

Desensitisation treatment/programs

Q99 Do you provide desensitisation treatment? (please choose all that apply)

- ☐ Insect venom (bee/wasp)
- ☐ Food
- ☐ Drug
- ☐ Desensitisation not provided

Q100 If you undertake desensitisation treatment to a food: For which foods do you provide desensitisation programs? (please choose all that apply)

- ☐ Peanut
- ☐ Milk
- ☐ Egg
- ☐ Other

If Other, please specify ____________

Allergy reaction management

Q101 Which adrenaline autoinjector device does your service issue? (tick as many as apply)

- ☐ EpiPen
- ☐ JEXT
- ☐ Emerade
- ☐ Other

If Other adrenaline autoinjector, please state which ____________

Q102 Which antihistamine do you routinely prescribe for food allergic reactions?

- ○ Cetirizine
- ○ Chlorphenamine (Piriton)
- ○ Other

If other antihistamine prescribed, please state which ____________

Q103 Where are your management plans for allergic reactions sourced from? (please choose all that apply)

- ☐ Do not issue management plans
- ☐ Locally designed
- ☐ BSACI
- ☐ From other centres
- ☐ Other

If Other, please specify ____________

Patient support and training

Q104 Where are your patient information sheets sourced from? (please choose all that apply)

- ☐ Do not provide any patient information sheets
- ☐ Locally designed
- ☐ Allergy UK
- ☐ Anaphylaxis Campaign
- ☐ From drug companies (Epipen/Emerade etc)
- ☐ From other centres
- ☐ Information sheets are not provided in the clinic
- ☐ Other

If Other, please specify ____________

Q105 Do you offer bespoke training to patients, parents and/or carers in the following in your clinic? (choose all that apply)?

- ☐ Do not offer any patient/parent training
- ☐ Training in the use of self injectable adrenaline
- ☐ Inhaler use
- ☐ Eczema management (use of emollients/steroids)
- ☐ Nasal spray/drop use
- ☐ Other

If Other, please specify ______

Service clinical governance & audit

The next couple of questions are about guidelines. Please answer honestly as to the extent you are aware of these and whether they have actually resulted in them being implemented in your paediatric allergy service.

Q106 Are you aware of the following NICE Clinical Guidelines?

	Yes - have read it and implemented it in our paediatric allergy service	Yes - have read it but not implemented it	Yes - but not read it	No
Food allergy in under 19s: assessment and diagnosis (CG116)	○	○	○	○
Anaphylaxis: assessment and referral after emergency treatment (CG134)	○	○	○	○
Drug allergy: diagnosis and management (CG183)	○	○	○	○

Q107 Do you have any comments you wish to make about the NICE Clinical Guidelines for food and drug allergy?

- ○ Yes
- ○ No

If Yes, please state comments ______

Q108 Are you aware of the RCPCH Allergy Care Pathways?

	Yes - have read it and implemented it in our paediatric allergy service	Yes - have read it but not implemented it	Yes - but not read it	No
Allergy care pathway for food allergy	○	○	○	○
Allergy care pathway for anaphylaxis	○	○	○	○
Allergy care pathway for venom allergy	○	○	○	○
Allergy care pathway for latex allergy	○	○	○	○
Allergy care pathway for urticaria, angio-oedema or mastocytosis	○	○	○	○
Allergy care pathway for asthma and/or rhinitis	○	○	○	○

Q109 Do you have any comments you wish to make about the RCPCH Allergy Care Pathways?

- ○ Yes
- ○ No

If Yes, please state comments ______

Q110 Do you hold a Multi-Disciplinary Team (MDT) meeting?

- ◯ Yes
- ◯ No

Q111 If you undertake a MDT meeting: How frequently are your MDT meetings held?

- ◯ Weekly
- ◯ Fortnightly
- ◯ Monthly
- ◯ Every two months
- ◯ Every three months
- ◯ Other

Q112 Is your service part of a Regional Paediatric Allergy Network?

- ◯ Yes
- ◯ No

If yes, which Network and how does the link work in practice? [ ]

Q113 Is your service formally linked to a tertiary Paediatric Allergy Centre

- ◯ Yes
- ◯ No

If yes, which tertiary Paediatric Allergy Centre and how does the link work in practice? [ ]

Q114 Do you offer paediatric allergy educational events? (please choose all that apply)

- ☐ For General Practitioners
- ☐ For colleagues in your hospital
- ☐ For members of the public
- ☐ Not offered
- ☐ Other

If Other, please specify [ ]

Q115 If you offer paediatric allergy educational events: How often do you offer paediatric allergy educational events

	Weekly	Fortnightly	Monthly	Every two months	Every three months	Every six months	Annually or less
For General Practitioners	◯	◯	◯	◯	◯	◯	◯
For colleagues in your hospital	◯	◯	◯	◯	◯	◯	◯
For members of the public	◯	◯	◯	◯	◯	◯	◯
Other	◯	◯	◯	◯	◯	◯	◯

Follow up arrangements in your service

Q116 Do you have a routine frequency of follow up for your paediatric allergy patients

- ◯ Yes
- ◯ No

Q117 What would your follow up policy be for the following patients in general (recognising that there will always be exceptions)

	Single consultation only	More frequent than annually	Annually	Every two years	Every three years or longer
Isolated nut allergy	○	○	○	○	○
Food allergy BUT NOT nut allergy	○	○	○	○	○
Nut and other food allergies	○	○	○	○	○
Pollen food/oral allergy syndrome only	○	○	○	○	○
Non-IgE type allergies (excluding FPIES)	○	○	○	○	○
FPIES	○	○	○	○	○
Complex allergy patients	○	○	○	○	○

Q118 It would be very helpful if you could describe in more detail your follow up policy, e.g. does your follow up policy vary according to patient age? If so, how?

Q119 Do you run an adolescent only clinic?

- ○ Yes
- ○ No

Q120 Do you run a transition clinic?

- ○ Yes
- ○ No

Q121 Does your hospital offer an adult allergy service?

- ○ Yes
- ○ No

Q122 What do you do with your patients when exceed the age threshold for your paediatric allergy service?

- ○ Discharge all of them back to primary care
- ○ Refer all of them to an adult allergy service
- ○ Refer some of them to an adult allergy service

Q123 If you refer them on: What adult services do you refer your patients on to?

- ☐ Adult allergy service
- ☐ Adult dermatology service
- ☐ Adult respiratory service
- ☐ Other

If Other adult services, please specify

Many thanks for completing the questionnaire

Q124 When our survey of all UK paediatric allergy services is complete would you like to receive a copy of the results?

- ◯ Yes
- ◯ No

If yes, please can you confirm your email address for us to send the survey results to: [ ]

Q125 Are you happy for your service to be identifiable by name for bench marking purposes?

- ◯ Yes
- ◯ No

Please would you now return the completed questionnaire to us.
(NB Your FOI department will require a completed copy for their records)

Options for how to send it back to us are:

1. Scan the completed questionnaire and email it to michael.perkin@nhs.net

2. Post it to:
Dr Michael Perkin
Room 1.27, 1st Floor, Jenner Wing
Population Health Research Institute
St George's, University of London
Cranmer Terrace
London, SW17 0RE
