# Supplementary material for: National Survey of United Kingdom Paediatric Allergy Services
Source: Clin Exp Allergy. 2022 Aug 3;52(11):1276–90. doi: 10.1111/cea.14198 (PMC9804618; doi:10.1111/cea.14198)
Supplement: Supplementary file 2 — Appendix S2 [file CEA-52-1276-s001.docx]

**Supplementary Appendix**

**National Survey of Paediatric Allergy Services**

**Authors:**

Rosy Wells,^1,2^ Cathy McKay,^2^ Nick Makwana,^3^ Deepan Vyas,^4^ Sophie Vaughan,^1^ Anne Christopher,^1^ Lucy Thomas,^1^ Misbah Primett,^1^ Lavanya Diwakar,^5^ Shamir Alvis,^6^ Michael R Perkin^1,2^

**Introduction**

In 2003, the Royal College of Physicians (RCP) published a seminal report “Allergy: the Unmet Need” in which it stated that “despite the epidemic proportions of the disease, the health service is failing to meet the most minimal standards of care – far less clinical governance.”^1^ The report noted the major shortage of allergy specialists, with only six fully staffed allergy clinics in the UK at that time (running more than five clinics per week) and 9 part time (one or two allergy clinics per week) adult and/or paediatric services provided by allergy specialists.^1^ A further 86 “part time” services were identified where services were offered by consultants in other specialities. The report stated that most of these “offer a limited spectrum of diagnostic and treatment facilities for allergy”.^1^ The limited opportunities for postgraduate clinical allergy training were felt to have contributed to knowledge of good allergy management in practice being therefore minimal or non-existent.^1^

The 2003 report was so scathing that it triggered a series of subsequent reports and reviews. The National Allergy Strategy Group (NASG), an alliance of the BSACI, the patient charities, Allergy UK and the Anaphylaxis Campaign and industry partners, formed in 2001 to highlight the need for allergy services and the inadequate care available for allergy patients at all levels in the NHS, produced a report in 2004 “An NHS Plan for Allergy - Making a Start”.^2^ The report calculated that with a UK birth rate per year of 650,000, an estimated minimum of 43,000 new cases of paediatric allergy requiring specialist advice could be expected to present each year.

In 2006 the Department of Health conducted “ A review of services for allergy”.^3^ The BSACI contributed three submissions for the review, one of which “The Patient Journey for Allergic Disease and a Model of Allergy Service within the NHS” incorporated a plan for a model of the NHS allergy service and optimal pathways of care within the proposed service.^4^ In the latter, reviewing services registered on the BSACI website,^5^ 26 out of 36 adult allergy clinics saw children, but only 8 involved a paediatrician. The adult allergy services had between 1 and 4 consultants and the services undertook between 1 and 6 outpatient clinics per week. There were 22 paediatric only allergy services, 14 were run by general paediatricians with an interest, 4 by paediatric allergists, 3 by paediatric immunologists and 2 by paediatric respiratory sub-specialists. They determined that the paediatric only services had a capacity to see 150 new patients a week (6750 per year assuming 45 clinics/year).^4^

In 2007 the House of Lords Science and Technology Committee produced a report “Allergy” in which it endorsed the Allergy: the Unmet Need recommendation that "a minimum of one specialist allergy centre should be established in areas equivalent to each of the former NHS regions, serving populations of five to seven million, to offer at least some local expertise for allergy sufferers, ….each centre should have as a minimum two adult allergy consultants, two paediatric allergy consultants supported by paediatric nurse specialists, two full-time nurse specialists, one half-time adult and one half-time paediatric dietician."^6^ In May 2009 a joint working party of the RCP and Royal College of Pathologists was established, supported by the BSACI, to collate evidence of progress in implementing the House of Lords recommendations.^7^ The report concluded that the actions taken to implement the original recommendations in full had been largely unsatisfactory.^7^

Subspecialty training in paediatric allergy has only been offered via the National Training Number (NTN) Grid since 2004 and training numbers in the subspecialty are far fewer than the number of paediatric allergists required to meet demand.^8^ Therefore, most paediatric allergy services have been developed, and are run by, general paediatricians. In 2012 the BSACI produced standards for paediatric allergy services in secondary care.^9^ These detail the specifications and requirements of a secondary care paediatric allergy service. However, there is no requirement for services to adhere to these standards and adherence has not been audited. In 2013, NHS England published service specifications for specialised allergy services for patients of all ages,^10^ as well as standards specific to paediatric allergy specialist centres.^11^ As with the secondary standards produced by BSACI, no adherence to these specifications have been audited and no accreditation system exists.

Treatments available within the field of paediatric allergy have diversified. Whilst immunotherapy has celebrated its centenary,^12^ sublingual immunotherapy, potentially more suitable for a paediatric allergy population, has experienced a resurgence of interest since 1986.^13^ However, it is recognised that the UK has an extremely poor record of offering allergen immunotherapy.^14^ In 2011 Vance *et al* reported the ‘unmet need’ for seasonal immunotherapy (SIT) through a survey of 20 NHS centres offering SIT to paediatric patients across the UK.^14^ 12 centres replied and 323 children had received SIT over 10 years i.e. 32 children per year. The authors then determined the percentage of eligible children that were being treated based on an estimate of 10% of 6-7 year olds and 15% of 13-14 year old having AR.^15^ The requirement for immunotherapy was extrapolated from a primary care survey of adult patients with AR where 27% were on maximal therapy and 15% remained symptomatic despite optimal medication and therefore eligible for SIT, i.e. 4% of AR patients.^16^ Vance determined that over 32,000 children aged 5-16 would be considered suitable for immunotherapy and that only 1% of affected paediatric patients had been treated over a ten year period.^15^

Reintroduction of allergenic foods into the diet has become a more formalised process with reintroduction regimens being published for various foods including egg.^17^ Oral immunotherapy (OIT) or food desensitisation has also existed for an almost identical time to immunotherapy, with the first case of egg OIT being described in a child in the Lancet in 1908.^18^ Whilst highly efficacious venom desensitisation is the universally accepted treatment for severe venom allergy, more contentious is the role of food desensitisation.^19,20^ Whilst cow’s milk and egg allergies are outgrown in the majority of children, the risk benefit analysis of speeding up natural tolerance acquisition through desensitisation continues to be debated. Potentially there is more value in desensitising children with specific food allergies that are likely to be life long, such as nuts and seeds.

With the launch of the BSACI Registry for Immunotherapy (BRIT) and the recent publication of its first report,^21^ our survey provides an opportunity to determine how comprehensive the recording of paediatric immunotherapy is on the registry.

The 2006 DH review of services for allergy resulted in their recommendation that the Department of Health consider the options for commissioning the development of NICE guidelines for allergy, and work with the Royal Colleges on guidance for referral and care pathways. Both NICE^22-24^ guidance and RCPCH pathways^25-30^ did subsequently emerge. We took the opportunity in this survey to ascertain the extent to which these are being utilised.

**Methods**

*Paediatric Allergy Service Questionnaire*

In 2017 consultants from the West Midlands Allergy Group performed a survey of 13 local hospitals to establish which services were being offered and to understand any differences between the hospitals. This survey was used as an initial template for our survey. Additional questions were based on the BSACI standards for secondary care,^9^ NHS specialist service specifications for paediatric allergy services^11^ and the BSACI SOP for skin prick testing.^31^ Other questions that the authors felt would provide valuable information about services across the UK were also included.

The questionnaire was created on Snap Surveys (Snap Ltd). The questionnaire included routing such that respondents only had to provide answers about services, investigations, or treatments that their service offered. The questionnaire underwent peer review prior to being sent out and was undertaken in partnership with the BSACI. Surveys could be completed either online or on paper. The latter could then be either scanned and emailed to the research team or returned to the postal address provided. Responses were recorded on to the Snap Survey software for online responses. Paper responses were entered into the SNAP database. Analysis of data was performed in Excel and Stata 15. Ethical approval was not required for this service evaluation. The responding hospital was identifiable but the person completing the questionnaire did not have to provide their identity. They were given the option of providing an email address if they wished to receive the full survey results.

The questionnaire included sections about the allergy investigations that each service undertakes including skin prick testing (SPT), intradermal testing and component testing. SPT questions included assessing how the procedure is performed based on BSACI^31^ and WAO^32^ recommendations. Questions included how the wheal size is determined – BSACI and WAO recommend measuring the largest diameter of the wheal and its perpendicular diameter. With regards to considering a skin prick test response to be positive, the BSACI consider a response of 3mm or more (than the negative control) to be a positive for older children and teenagers and any size response to be positive for babies and young children. The WAO solely uses the 3mm or more than the negative control threshold. The WAO further recommends that the type of lancet used to do the skin prick testing be taken into account in setting the threshold for determining an allergen positive response, as some lancets are more likely to generate a positive response to the negative control solution, presumed to be related to the amount of trauma a specific lancet induces. Hence for some lancets it proposes that a response of 3mm or more to an allergen be deemed positive and for others a response of more than 3mm to the allergen.^32,33^ Both WAO and BSACI do not propose subtracting a negative control response, when positive, from the allergen responses. The BSACI states that skin prick testing is invalid if the negative control is positive, unless the allergen response is 3mm larger than the negative control response. The WAO state to “avoid devices or techniques that produce a negative control >3 mm wheal and >10 mm flare, due to the possibility of producing a false positive reaction”.^32^ If pseudopods are present both the WAO and BSACI do not recommend included them in the measurement of the largest diameter. With regards to the size of the positive control response, the WAO indirectly stipulate that the response should be 3mm or more for the testing to be valid.

An updated standard operating procedure for paediatric allergy SPT was published in 2019 by the BSACI Nurses in Allergy Committee^31^ and a position paper on IgE allergy diagnostics and other relevant tests in allergy by the World Allergy Organization in 2020.^32^ Recommendations from both documents were incorporated in the questionnaire. Detailed questions were included about the provision of challenges for food and or drugs.

Management questions encompassed use of reintroduction ladders, desensitisation, allergic reaction management and patient support and training and follow up arrangements beyond paediatric care. Governance issues included knowledge of NICE^22-24^ and RCPCH^25-30^ allergy related guidance, and service links with wider allergy networks.

**Results**

***Location, structure and staffing***

*Links with Primary Care*

Seven per cent (11) of services offer a paediatric allergy service in primary care, with staff from the trust going into primary care. In 4% (6) of services, GPs have been trained to provide a paediatric allergy service in primary care. Sixteen percent (25) of services reported ‘other’ provision of allergy services in primary care, of which 15 reported providing training/teaching in primary care. Four services provide an advice service for GPs and two services hold regular MDTs with primary care to discuss patients. One service has a GP with specialist interest in allergy providing a service at the hospital site, one service has a nurse led allergy clinic in primary care, and a further service is currently training GPs to provide an allergy service in primary care.

*Medical Staffing*

Given the very variable proportion of consultant working time that might be linked specifically to providing paediatric allergy services in a secondary level allergy service, the absolute number of consultants that were declared to be providing paediatric allergy services in this setting (median 2, mean 3.6, range 0-16) is less useful than the number of consultant whole time equivalents (WTE) providing services specifically to paediatric allergy patients (median 0.5, mean 1.3, range 0-14). However, the upper limit of the range indicates that there were clearly issues with how the latter too had been interpreted with some secondary services not restricting the WTE provision to that for paediatric allergy patients.

In contrast, consultant configuration responses for tertiary services are unlikely to be subject to the same issues of interpretation. Tertiary paediatric allergy services are provided by an absolute number of consultants ranging from 1-14, median 4, mean 4.5. The median WTE is 1.5, mean 1.7, range 0.2-6.1. The total number of consultants contributing to seeing paediatric allergy patients across all services in the UK is 575. There are also 2 services across the country with no consultants, both run by associate specialists.

In both secondary and tertiary level services, general paediatricians with a subspecialty interest in allergy (with less than 50% of time specifically with paediatric allergy patients), are the most frequently represented clinicians seeing paediatric allergy patients. Consultant subspecialists are present in 54% (15/28) of tertiary services and 7% (9/126) of secondary services. Amongst paediatric allergy services with general paediatricians with a subspecialty interest contributing to seeing paediatric allergy patients, allergy is the most frequently declared specific interest, followed by respiratory and about one in five declaring dermatology or gastroenterology as their interest. For paediatric consultant sub-specialists, the same pattern is seen with allergy predominant. However, there is significant representation of immunology subspecialists in tertiary services. Medical staff in tertiary services are more likely to have obtained formal qualifications in allergy.

Eighty two percent of services (125/153) have a lead for their paediatric allergy service. One third (52/154) of services report that all consultant staff contributing to their paediatric allergy service have a minimum of 2PAs in their job plans designated to paediatric allergy. Eighty one percent ( 120/149) report that at least one member of staff involved with paediatric allergy patients is a member of the BSACI.

Fifty-five per cent ( 84/153) of services have registrars or clinical research fellows who regularly attend clinics where paediatric allergy patients are seen. Of these, 44% (37/84) are seeing patients independently and 56% (47/83) sit-in on clinics.

*Nursing Staffing*

In secondary services the absolute number of nurses that contribute to looking after paediatric allergy patients ranges from 0 to 10 individuals, median 2, mean 2.1. In tertiary services, the range is from 0 to 14 individuals, median 3, mean 4.3. The number of WTE nurses in secondary services is 0 to 4, median 0.8, mean 0.9 and in tertiary services 0.2 to 11, median 2.0, mean 2.7. A total of 363 nurses contribute to seeing paediatric allergy patients across the 144 services in the UK who provided data for this question.

The Band distribution of nurses working in the paediatric allergy service is similar for Bands 5 to 7 between secondary and tertiary paediatric allergy services: Band 5 26% (63/244 nurses) vs 26% (30.5/119) respectively; Band 6 42% (103/244) vs 42% (50/119); and Band 7 26% (63/244) vs 26% (32/119). In contrast, the 19 Band 8 nursing roles are predominantly in tertiary services and consisted of: Nurse Consultant (6), Advanced Nurse Practitioner (4), Clinical Nurse Specialists (6), matron (2) and allergy nurse manager (1).

Formal allergy training status of nursing staff is similar comparing secondary and tertiary level services: all nurses have formal training 35% (36/104) versus 37% (10/27) respectively; some nurses have formal training 43% (45/104) versus 59% (16/27). However secondary services are more likely to have no nurses who had had formal training in allergy: 22% (23/104) versus 4% (1/27).

Independent nurse clinics are held in 60% (78/129) of services: 85% (22/26) of tertiary and 54% (56/103) of secondary services. At these clinics, 45% (35/77) see new referrals, 75% (58/77) see follow up patients, 86% (66/77) see patients for SPT or training and 21% (16/77) see immunotherapy patients independently.

*Dietetic provision*

Ninety two percent (115/125) of secondary services and all (28/28) tertiary services have dietetic support; 18% (27/153) of services have dietetic support at all paediatric allergy clinics, 30% (46/153) at some and 46% (70/153) report a dietician is not present in clinic but sees patients after a referral from the paediatric allergy service. Dietetic support in secondary services specifically for paediatric allergy ranges from 0 to 2.5 WTE, median 0.1, mean 0.2 and in tertiary services ranges from 0 to 2.2 WTE, median 0.5, mean 0.6. Dieticians are undertaking independent clinics seeing new paediatric allergy patients in 30% (34/112) and 41% (11/27) of secondary and tertiary services respectively.

*Allergy Clinics*

Ten percent (16/154) of services see paediatric allergy patients exclusively in general clinics, 57% (88/154) in dedicated paediatric allergy clinics and 32% (50/154) in both. Exclusively paediatric allergy clinics are more common in tertiary services (median 6 per week, range 1-59) than secondary services (median 2 per week, range 1-8). The converse is true for general paediatric clinics where paediatric allergy patients are seen, with more such clinics in secondary services (median 4 per week, range 1-20) than in tertiary services (median 2.5 per week, range 1-12).

Respondents estimated the proportion of general paediatric clinic consultations that are related to paediatric allergy problems as between 1 and 75% (median 15%). Whilst the range reflects the difficulty in estimating such a figure, the median implies that one in six general paediatric consultations is allergy related.

Service capacity is heavily skewed towards smaller services. 58% of services see 10 or less new allergy patients per week and 28% see 5 or less per week. Within secondary level services the figures are higher: 66% and 33% respectively. Secondary care services (119) see a median of 8 new patients per week (range 1-30) and 10 follow up patients (range 1-60) (Supplementary Figure 1). Assuming 45 clinics per year this yields a capacity of 51,000 new patients and 64,400 follow up patients per year. For tertiary services, the median figures are 21 new patients per week (range 4-171) (34,600 per year) and 30 follow up patients (range 6-154 patients) (47,000 per year). Hence total capacity is 85,600 new paediatric allergy appointments and 111,400 follow up appointments per year in the UK.

The median waiting time is 3.0 months (range 0-24 months) for new patient appointments and 4.0 months (range 0–24 months) for follow up. There are two services reporting waiting times of >12 months for new patient appointments, both in Northern Ireland.

Joint clinics are offered by 21% ( 32/154) of services: dermatology 56% (18/32), gastroenterology and respiratory both 38% (12/32). Nine services offer joint clinics with other specialities: ENT/ophthalmology (5), tertiary consultant clinics (2), psychology (1), immunology (1) and anaesthetics (1).

*Structure of clinics*

Both morning clinics (148) and afternoon clinics (111) range from 1 to 5 hours (median 4 hours). Four percent (6) of services offer evening clinics seeing paediatric allergy patients.

The median consultant consultation duration is 30 minutes for a new patient and 20 minutes for a follow up. However, for both secondary and tertiary services, the most common appointment duration permutation is 30 minutes for a new patient and 15 minutes for a follow up (Supplementary Figure 2). The median duration of new patient appointments for associate specialists, allergy nurse specialists, specialist registrars and dieticians is the same as consultants at 30 minutes (range 15-60 mins). Median duration for follow up appointments for associate specialists and dieticians is the same as consultants at 20 minutes, but for allergy nurse specialists and registrars is longer at 30 minutes.

Clinic templates for number of new and follow up patients are broadly similar for morning and afternoon clinics. However, there is a notable difference in configuration for clinics seeing exclusively paediatric allergy patients with the most common configuration being 4 new patients and 4 follow ups per clinic, as compared to general paediatric allergy clinics where allergy patients are seen where the most common configuration is 4 new patients and 6 or 8 follow up patients (morning general clinics) and 3 or 4 new patients with 6 follow up patients (afternoon general clinics) (Supplementary Figure 3).

*Allergy clinic coding*

55% (85/154) of respondents declared they know how paediatric allergy outpatients are coded: 52% (44/85) as ‘420 Paediatrics’, 27% (23/85) as ‘255 Paediatric Clinical Immunology and Allergy’ and 9% (8/85) as ‘317 Allergy’. The 255 code is much more commonly used in tertiary services (60%, 12/20) as compared with secondary services (17%,11/65).

Twenty seven percent (42/154) of respondents know the approximate reimbursement price for patients seen in allergy services. Mean reimbursement costs for tertiary services for new patient, appointments, follow up appointments and day cases are higher than in secondary services, but a wide range is reported (Supplementary Table 1). Where the coding of outpatient clinics is known, reimbursement costs for a new patient consultation within each code are more consistent. The code ‘317 Allergy’ attracted the highest tariff, followed by ‘255 Paediatric Clinical Immunology and Allergy’ and then ‘420 Paediatrics’ (Supplementary Table 1).

***Investigations***

*Skin Prick Testing*

SPT is performed on the same day as the outpatient clinic appointment in 93% (111/120) of secondary services and 96% (27/28) of tertiary services. Testing is largely performed by nursing staff and also by a minority of consultants, but different patterns were seen in tertiary and secondary services with the latter having more consultants undertaking testing and less nurses compared to tertiary services. Dieticians undertake testing in 6% (9/148) of services, associate specialists in 4 services, specialist registrars in 3 services and a laboratory technician in 1 service. Other staff reported to perform skin testing are health care assistants (7) and ENT staff (1).

Nearly all centres (99%, 146/148), have resuscitation facilities immediately available for SPT. SPT are performed to foods using commercial SPT solutions in 99% of services (147/148), to fresh foods (prick prick testing) in 94% (139/148) and to aeroallergens in 94% (139/148) of centres. Latex, bee/wasp venom and drug SPT are offered in fewer services with 74% (109/148), 31% (46/148) and 28% (41/148) offering these respectively. Venom and drug SPT are predominately done in tertiary services.

The commercial solutions most commonly used for SPT are: Soluprick (ALK) (84), Allergy Therapeutics (68), Bio-Diagnostics (Immunotek) (51), Diagenics (Allergopharma) (101) and Lofarma (11).

Of the services performing SPT to whole foods, 93% (129/138) of services provide prick prick testing to foods brought in by patients/parents. Fresh cow’s milk is tested by 62% (85/138), sesame (tahini) by 56% (77/138), raw egg by 30% (42/138) and whole nuts by 41% (56/138) of services. Whole nuts used for prick prick testing vary significantly across services: cashew is used in 17 services, walnut 17, pecan 13 and pistachio 13. Some services report that they use less common nuts (macadamia, pecan) in the absence of having commercial skin prick test solutions for these nuts. Eleven services (8 secondary, 3 tertiary) use whole nuts for every nut tested.

In services performing SPTs to sesame as tahini, 45% (34/76) of these services test to commercial sesame solution at the same time. For those using fresh cow’s milk, 73% (59/81) also test to commercial solution of cow’s milk and for raw egg, 83% (33/40) also test to commercial egg solution. In services using whole nuts for SPTs, 42% (21/50) also test using the commercial solution of the nut at the same time.

*Individual practices for skin prick testing*

When measuring SPT wheal size, 68% (100/147) of respondents measure the largest diameter of the wheal and 32% (47/147) measure the mean of the largest diameter of the wheal and its perpendicular diameter (BSACI and WAO recommendation). 88% (130/147) consider a skin prick test size of 3mm or more (than the negative control) a positive response (BSACI older children and teenagers and WAO recommendation), and the remaining 12% consider any response to be positive (BSACI consider any size response positive for babies and younger children).

When the negative control is positive, 37 % (55/148) of respondents deduct the size of the negative control from the allergen responses (not proposed by either the WAO or BSACI guidelines). If pseudopods are present, their diameter is included in the measurement of the largest diameter by 21% (30/146) of respondents (not recommended by BSACI or WAO). 80% (118/148) of respondents agree that the positive control must exceed a threshold size for the test to be valid (WAO indirectly stipulate 3mm or more), and 82% (90/110) of those individuals reported that the threshold was 3mm.

***Diagnostics***

*Food challenge configuration*

Open food challenges predominantly take place on day wards. Dedicated challenge units are rare and almost exclusively in tertiary centres (1 secondary service and 6 tertiary services). A minority of open food challenges are undertaken in outpatients (9 secondary services and 3 tertiary). Open food challenges on inpatient wards were much more likely to occur in secondary services. Locations where supervised feeds were undertaken were broadly similar, with the exception of outpatient departments which were used significantly more frequently for both secondary but particularly tertiary service supervised feeds.

Two secondary level services report not having resuscitation facilities available for challenges as opposed to the other 140 services that do.

The median number of challenges undertaken in one session is: secondary 2, IQR 1-2, range 1-8 and tertiary 4, IQR 2-5, range 1-12. When asked to describe the configuration of challenge sessions with more than one patient attending, 36% (25/70) of secondary services reported patients all arriving at the same time compared with 15% (4/27) of tertiary services. In contrast, tertiary services were more likely to use a staggered arrival regimen: 48% (13/27) compared with secondary services 29% (20/50).

The most common ratio of number of patients undertaking food challenges per supervising staff member was 1:1 for 45% of services (60/134). 2:1 was used for 43% (57/134), but 6% (8/134) had a ratio of 4:1 and one service 5:1. For services stratifying their challenges by risk status, for high risk challenges a 1: 1 ratio was used more frequently in 82% (58/71) of such services. 16% (11/71) had a high risk challenge ratio of 2:1. Two secondary services undertook high risk challenges with a ratio of 4:1. In addition to the change in patient to staff ratio, those undertaking challenges designated high risk were asked to describe how the challenge configuration was different to a standard challenge. Responses including using additional stages, insertion of an intravenous line, prolonged observation period and having more senior/experienced supervision of the challenge. Those services undertaking supervised feeds were more likely to use higher patient staff ratios for these: 3:1 10% (5), 4:1 18% (9) and 5:1 in one service.

*Food challenge staffing*

Food challenges in secondary level services were most frequently supervised by paediatric nurses, followed by allergy nurse specialists. Tertiary service challenges were most commonly undertaken by an allergy nurse specialist but use of a consultant or a specialist registrar was more common than in secondary services. Most food challenges being undertaken are now nurse led, more frequently in tertiary (93%, 25/27) than secondary (78%, 84/108) services.

Specific cover arrangements for nurse led challenges was varied: Junior doctors provide cover for clerking, consent and treatment of reactions in 27% of services (29/108); junior doctors present on unit but not directly involved with challenges in 16% (17/108); doctor providing paediatric allergy services not present but scheduled to be available if needed in 13% (14/108); junior doctor not present but specifically scheduled to be available if needed in 4% (4/108); general paediatric on call team specifically scheduled to be available if needed in 10% (11/108); no specific cover arranged - general paediatric on call team could be called if needed in 15% (16/108) and 16% (17/108) had other arrangements. The online questionnaire allowed only one of the above options to be selected, however those respondents completing a paper copy of the questionnaire often selected more than one option for this question, suggesting that the cover arrangements are more complex than the options given.

**Discussion**

In the Allergy the Unmet Need report, the BSACI compiled a list of allergy services (paediatric and/or adult) existent in 2003 and identified 6 services with a “whole time” allergy specialist (defined as running more than 5 allergy clinics per week), 9 services provided part time by allergists (providing “one or two allergy clinics per week”, and 86 part time services offered by consultants in other specialities “most of whom offer a limited spectrum of diagnostic and treatment facilities for allergy”. The British Allergy Foundation identified an additional 15 services run by NHS consultants who were not BSACI members. The Report derived a WTE figure for consultant allergist-led service provision by assigning 1 WTE to each of the 6 whole time services, 0.3 WTE for each of the part time allergist services and 0.1 WTE for each of the 101 (86+15) part time other services. This generated a figure of 17.3 WTE consultant allergist led clinics for the UK. From our results there are now 19 services undertaking more than 5 paediatric allergy clinics per week, 6 of whom designate themselves as secondary level services. There are twenty services with at least one paediatric allergist: 14 tertiary services and 6 secondary services. Of these twenty services with paediatric allergy specialists, eight are services undertaking five or less paediatric allergy clinics per week (3 secondary services and 5 tertiary services). Furthermore, two services with paediatric allergy subspecialists only see paediatric allergy patients within general paediatric clinics (both secondary services). This highlights the shortcomings in designating service provision based on an arbitrary ‘clinics per week’ threshold. Determining WTE provision within tertiary services is likely to be accurate and the total WTE provision within the 28 tertiary services is 47.75 WTE. Additional to this is the WTE equivalents provided in the 126 secondary services. Unfortunately a minority of respondents in secondary level services either misinterpreted or found it too difficult to translate the absolute number of consultants providing a paediatric allergy service into a whole time equivalent figure specifically related to the provision of paediatric allergy services and gave either the same figure (e.g. 14 general consultants and 14 WTE), or a figure that seemed in excess of the activity as based on the number of new and follow up consultations for paediatric allergy patients being undertaken per week. However, to assign a WTE of 0.1 to all the secondary services would significantly underestimate provision given that the secondary services include the 6 undertaking more than five paediatric allergy clinics and a further 10 undertaking five paediatric allergy clinics per week.

Notable is how prevalent is the use of whole foods for SPT, particularly fresh cow’s milk and tahini. Many services undertake SPT with the commercial skin prick test solution at the same time as skin testing with whole foods, particularly fresh cow’s milk and tahini. We did not collect data on the reasons for using fresh foods but it would have been interesting to investigate how this is used in clinical practice. Whilst using fresh foods increases the sensitivity of SPT (from 40% to 81.3% In one study),^34^ this inevitably comes at the expense of decreased specificity and the corollary of this, an increased false positive rate. One therefore has to be in a position to have sufficient challenge capacity to distinguish between the false positive results that fresh foods generate and those with genuine food allergies.

References

1. Royal College of Physicians. *Allergy: the unmet need. A blueprint for better patient care.* London: Royal College of Physicians of London,;2003. 1860161839

2. National Allergy Strategy Group. *An NHS Plan for Allergy - Making a Start.* 2004.

3. Health: Do. *A review of services for allergy.* 2006m.

4. British Society of Allergy and Clinical Immunology. *The Patient Journey for Allergic Disease and a Model of Allergy Service within the NHS.*  04/2006 2006.

5. British Society of Allergy and Clinical Immunology. Find a clinic. https://www.bsaci.org/workforce/find-a-clinic/. Accessed 12/03/2021.

6. House of Lords Science and Technology Committee. 6th Report of Session 2006-07. *Allergy. Volume I: Report.* London2007.

7. Royal College of Physicians. *Allergy services: still not meeting the unmet need.* London2010.

8. Wardlaw AJ, Cale, C.M., Corrigan, C.,Cox, H., Durham, S.R., Farooque, S., Frew, A.,Kumararatne, D.S.,Stroud, C.,Thomas, M. The relationship between allergy and clinical immunology. Report of a Working Group convened by the BSACI,. 2005.

9. British Society of Allergy and Clinical Immunology. Paediatric Committee. Standards for paediatric allergy services in secondary care. In:2012.

10. NHS England. NHS Standard Contract for Specialised Allergy Services (All Ages). Section B Part 1 - Service Specificiations. In:2013.

11. NHS England. NHS Standard Contract for Paediatric Medicine: Specialised Allergy Services. Schedule 2 -The Services A. Service Specificiations. In:2013.

12. Durham SR, Nelson H. Allergen Immunotherapy: A Centenary Celebration. *World Allergy Organization Journal.* 2011;4(6):104-106.

13. Frew AJ. Hundred years of allergen immunotherapy. *Clinical & Experimental Allergy.* 2011;41(9):1221-1225.

14. Vance GHS, Goldring S, Warner JO, et al. A national audit of pollen immunotherapy for children in the United Kingdom: patient selection and programme safety. *Clinical & Experimental Allergy.* 2011;41(9):1313-1323.

15. Asher MI, Montefort S, Bjorksten B, et al. Worldwide time trends in the prevalence of symptoms of asthma, allergic rhinoconjunctivitis, and eczema in childhood: ISAAC Phases One and Three repeat multicountry cross-sectional surveys. *Lancet.* 2006;368(9537):733-743.

16. White, Smith, Baker, Davis, Frew. Symptom control in patients with hay fever in UK general practice: how well are we doing and is there a need for allergen immunotherapy? *Clinical & Experimental Allergy.* 1998;28(3):266-270.

17. Clark AT, Skypala I, Leech SC, et al. British Society for Allergy and Clinical Immunology guidelines for the management of egg allergy. *Clinical & Experimental Allergy.* 2010;40(8):1116-1129.

18. Schofield AT. A case of egg poisoning. *Lancet.* 1908;1(716):67313-67310.

19. Duca B, Patel N, Turner PJ. GRADE-ing the Benefit/Risk Equation in Food Immunotherapy. *Current Allergy and Asthma Reports.* 2019;19(6):30.

20. Chu DK, Wood RA, French S, et al. Oral immunotherapy for peanut allergy (PACE): a systematic review and meta-analysis of efficacy and safety. *Lancet.* 2019.

21. Erlewyn-Lajeunesse MW, Peter;Kinsman, Robin;. *The British Society for Allergy & Clinical Immunology First Registry for Immunotherapy Report.* 2020.

22. National Institute of Clinical Excellence. *Food allergy in under 19s: assessment and diagnosis (CG116).* 2011.

23. National Institute of Clinical Excellence. *Anaphylaxis: assessment and referral after emergency treatment (CG134).* 2011.

24. National Institute of Clinical Excellence. *Drug allergy: diagnosis and management (CG183).* 2014.

25. Royal College of Paediatrics and Child Health. *Allergy Care Pathways for Children: Food Allergy.* 2011.

26. Royal College of Paediatrics and Child Health. *Allergy Care Pathways for Children: Anaphylaxis.* 2011.

27. Royal College of Paediatrics and Child Health. *Allergy Care Pathways for Children: Venom Allergy.* 2011.

28. Royal College of Paediatrics and Child Health. *Allergy Care Pathways for Children: Latex Allergy.* 2011.

29. Royal College of Paediatrics and Child Health. *Allergy Care Pathways for Children: Urticaria/Angio-oedema/Mastocytosis.* 2011.

30. Royal College of Paediatrics and Child Health. *Allergy Care Pathways for Children: Asthma/Rhinitis.* 2011.

31. British Society of Allergy and Clinical Immunology. Nurses in Allergy Committee. Standard Operating Procedure: Paediatric Allergy Skin Prick Testing. 2019.

32. Ansotegui IJ, Melioli G, Canonica GW, et al. IgE allergy diagnostics and other relevant tests in allergy, a World Allergy Organization position paper. *World Allergy Organization Journal.* 2020;13(2):100080.

33. Bernstein IL, Li JT, Bernstein DI, et al. Allergy Diagnostic Testing: An Updated Practice Parameter. *Annals of Allergy, Asthma & Immunology.* 2008;100(3, Supplement 3):S1-S148.

34. Rancé F, Juchet A, Brémont F, Dutau G. Correlations between skin prick tests using commercial extracts and fresh foods, specific IgE, and food challenges. *Allergy.* 1997;52(10):1031-1035.

**Supplementary Table 1.** Approximate reimbursement costs by consultation type and, for new patient appointments, by clinic code

|  | **Secondary** | | | **Tertiary** | | |
| --- | --- | --- | --- | --- | --- | --- |
| **Consultation type** | **n** | **Mean (£)** | **Range (£)** | **n** | **Mean (£)** | **Range (£)** |
| New Patient | 26 | 242 | 140-397 | 11 | 295 | 215-380 |
| Follow up | 25 | 152 | 80-373 | 11 | 211 | 140-380 |
| Day Case | 9 | 462 | 221-700 | 10 | 610 | 300-1013 |
| **Clinic code** |  |  |  |  |  |  |
| 255 PCIA | 4 | 277 | 240-312 | 7 | 284 | 215-367 |
| 317 Allergy | 1 | 373 | 373 | 2 | 319 | 271-367 |
| 420 Paediatric | 13 | 222 | 159-250 | 3 | 259 | 252-271 |
| Don't know | 6 | 244 | 140-397 | 2 | 312 | 282-341 |
| Other | 2 | 233 | 216-249 | 3 | 333 | 252-380 |

PCIA- Paediatric Clinical Immunology and Allergy

**Supplementary Figure legends**

**Supplementary Figure 1.** Number of new (Panel A) and follow up (Panel B) paediatric allergy consultations per week by service level

**Supplementary Figure 2.** New and follow up patient consultation time in secondary (n=125) and tertiary (n=24) services for consultant paediatric allergy appointments

**Supplementary Figure 3.** Number of new and follow up patients seen in morning and afternoon clinics in general paediatric and specialist paediatric allergy clinics

**Supplementary Figure 4.** Follow up frequency undertaken for specific patient conditions. Respondents were given a series of hypothetical patient conditions and asked to report how frequently they would follow up such a patient. We acknowledged that this might alter subject to a number of cofactors.

**Supplementary Figure 1.** Number of new and follow up consultations per week by service level

**Supplementary Figure 2.** New and follow up patient consultation time in secondary (n=125) and tertiary (n=24) services for consultant appointments

**Supplementary Figure 3.** Number of new and follow up patients seen in morning and afternoon general paediatric and specialist paediatric allergy clinics

**Supplementary Figure 4 Follow up frequency**
